# Perceived norms, personal agency, and postpartum family planning intentions among first-time mothers age 15–24 years in Kinshasa: A cross-sectional analysis

**DOI:** 10.1371/journal.pone.0254085

**Published:** 2021-07-09

**Authors:** Anastasia J. Gage, Francine E. Wood, Pierre Z. Akilimali

**Affiliations:** 1 Global Community Health and Behavioral Sciences, Tulane University School of Public Health and Tropical Medicine, New Orleans, Louisiana, United States of America; 2 Kinshasa School of Public Health, University of Kinshasa, Kinshasa, Democratic Republic of Congo; University of Salamanca, SPAIN

## Abstract

Unintended pregnancy is an important global health problem and frequently occurs during the immediate postpartum period. However, few studies have examined postpartum family planning (PPFP) intentions among adolescent girls and young women. This study assessed whether perceived norms and personal agency predicted PPFP intentions among first-time mothers age 15–24 in Kinshasa, the Democratic Republic of Congo. Data were derived from the 2018 Momentum Project baseline survey. Analysis was based on 2,418 nulliparous pregnant women age 15–24 who were approximately six months pregnant with their first child in six health zones of Kinshasa. Overall PPFP intentions were low and ten to thirteen percent of women stated they were very likely to discuss PPFP next month with (a) their husband/male partner and (b) a health worker, and to (c) obtain and (d) use a contraceptive method during the first six weeks following childbirth. The results of multivariable linear regression models indicated that the PPFP intention index was predicted by description norms, perceptions of the larger community’s approval of PPFP, normative expectations, perceived behavioral control, self-efficacy, and autonomy. Rejection of family planning myths and misconceptions was also a significant predictor. Interaction terms suggested that the association of normative expectations with PPFP intentions varied across ethnic groups and that the positive association of injunctive norms with PPFP intentions was significantly increased when the larger community was perceived to disapprove of PPFP use. Normative expectations and PPFP-related self-efficacy accounted for two-thirds of the variance in PPFP intentions. The results suggested that understanding different normative influences may be important to motivate women to use contraception in the immediate postpartum period. In addition to addressing institutional, individual, and social determinants of PPFP, programs should consider integrating norm-based and empowerment strategies.

## Introduction

Postpartum family planning (PPFP)—defined as the prevention of unintended pregnancy and closely spaced pregnancies through the first 12 months following childbirth—is widely recognized as a key strategy for reducing unmet need for contraception [1], and can improve the survival chances of mothers and children. Contraceptive use has the potential to avert 440,000 neonatal deaths, 473,000 child deaths, and 564,000 stillbirths globally if 90 percent of unmet need for contraception were satisfied [2]. Short birth-to-pregnancy intervals of less than 12 months are the riskiest for the mother and baby and are associated with adverse pregnancy outcomes, such as preterm birth, low birth weight, and small for gestational age [3]. The risks of child mortality are highest for a birth-to-conception interval less than six months and second highest for an interval of 6–11 months. An analysis of Demographic and Health Survey (DHS) data from 45 countries found that if all couples waited 36 months to conceive again, under-five mortality would decrease by 26 percent [4]. As rapid repeat pregnancies (that is, pregnancies occurring within 12–18 months after delivery) can occur if women are unsuccessful at initiating contraceptive use after delivery, the examination of PPFP intentions is a public health imperative.

A recent systematic review and meta-analysis of studies in low- and middle-income countries indicated that uptake of PPFP was low, about 41 percent overall, but was much lower in West Africa. Perception of low pregnancy risk due to breastfeeding and postpartum amenorrhea, fear of method side effects, inadequate family planning (FP) counseling during the prenatal and postnatal period, and concern about partner disapproval were associated with non-use of PPFP [5]. Male partner approval was positively associated with contraceptive intentions among postpartum women in Ethiopia [6], while antenatal counseling had a positive effect on postpartum contraceptive intentions but not on use in northern Tanzania [7]. In one study, the odds of using a modern FP method at one-year postpartum were significantly higher among women who reported they intended to use FP immediately postpartum than among those with no stated intention [8].

Although complications from pregnancy and childbirth are the leading causes of death to adolescent girls and young women worldwide [9], with pregnancies occurring in the postpartum period being associated with increased risks of adverse maternal and newborn health outcomes, there is a dearth of studies on PPFP intentions and use among adolescent girls and young women in sub-Saharan Africa. The value of analyzing PPFP intention among FTMs age 15–24 cannot be overstated. Every year, an estimated 10 million unintended pregnancies occur to adolescent girls age 15–19 in developing countries [10]. The World Health Organization recommends a minimum interval of at least 24 months after a live birth and six months after a miscarriage or abortion before attempting the next pregnancy [11,12]. However, knowledge gaps, limited life experiences, and belief in FP myths and misconceptions may constrain PPFP intentions and use among young mothers who want to avoid a subsequent pregnancy [13]. Community and religious norms that prohibit nonmarital sex and childbearing may lead to labelling, gossip, marginalization, mistreatment, low self-esteem, internalized shame, and stigma among unmarried pregnant and childbearing adolescents [14,15]. In addition, adolescent girls and young women may lack the agency and autonomy to exercise personal control over and negotiate PPFP use [16]. Postpartum women and those aged less than 20 years tend to have some of the highest levels of unmet need for FP [17]. An analysis of DHS data for 57 countries showed that in 2005–2013, 62 percent of women had an unmet need for contraception right after delivery and 43 percent, after six months of amenorrhea [18].

**Fig 1:**
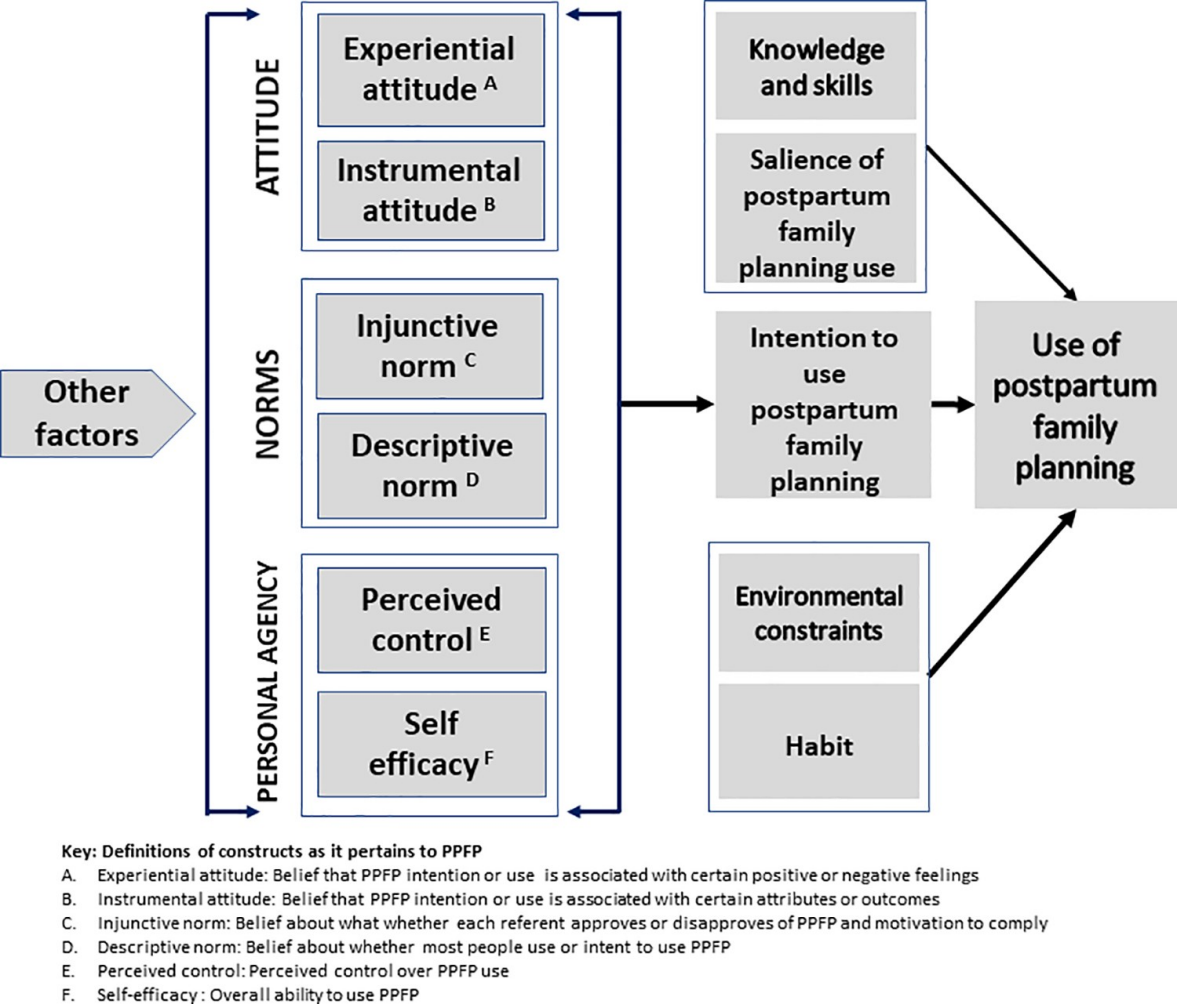
Integrated behavioral model of postpartum family planning use.

To understand behavioral intentions and health behavior, and plan for health promotion activities, theories such as the Theory of Planned Behavior (TPB) and the Theory of Reasoned Action are sometimes used [19–21]. The Integrated Behavioral Model (IBM), which includes constructs from the TPB and the Theory of Reasoned Action [22], provides added value for examining PPFP intentions among adolescent girls and young women. As depicted in Fig 1, in the IBM, behavioral intention is determined by three main factors: (1) attitude toward the behavior, defined as a person’s overall favorableness or un-favorableness toward performing the behavior, which has both affective and cognitive dimensions; (2) perceived norm which reflects beliefs about what others think one should do and motivation to comply (injunctive norms) and perceptions about what others in one’s social or personal networks are doing (descriptive norms); and (3) personal agency, which reflects both self-efficacy and perceived behavioral control. The IBM postulates that the influence of socioeconomic and demographic variables on intentions and behavior is indirect and operates largely through the model constructs [22,23].

The IBM has been used to understand intention and behavior related to condom use, physical activity, breast cancer screening, male circumcision, and substance use prevention but, to date, the model has not been applied as a framework for identifying key attitudinal, normative and control beliefs influencing PPFP intention and use. Research on the influence of attitudes on contraceptive intention among adolescent girls and young women has highlighted the role played by misinformation, misperceptions, and incomplete information about contraceptive methods in fostering negative attitudes towards contraceptive use and lowering contraceptive intentions [24,25]. FP myths, misperceptions, and exaggerated side effects, such as “people who use contraceptives end up with health problems,” “contraceptives are dangerous to women’s health” and “contraceptives can harm your womb”, as well as a perception that contraceptives may negatively affect future fertility have been demonstrated to be highly prevalent at the individual and community levels in sub-Saharan African countries [26] and among adolescents in the United States [24], leading to negative attitudes towards modern contraceptive use. Studies on the effect of women’s attitudes on PPFP intentions are scarce. In general, positive attitudes towards FP have been associated with postpartum contraceptive use among expectant inner-city women in the United States [27], use of long-acting and permanent contraception in Southern Ethiopia [28] and contraceptive use in Nepal [29], while unfavorable attitudes towards modern contraceptive methods has been found to impede demand for contraception and to be significantly associated with unmet need for FP [30]. Similarly, positive attitudes towards condom use had significant direct effects on condom use intentions and/or actual use [31–35].

Regarding the influence of normative beliefs on intention and actual behavior, applications of the IBM or TPB constructs have revealed that, in general, favorable injunctive norms are positive predictors of contraceptive intention and use. For example, in the Democratic Republic of Congo (DRC), Costenbader et al. [36] found that intention to use a modern contraceptive method increased by 0.6 with every unit increase in in injunctive FP norms and that injunctive norms about community approval of gender-equitable roles in childcare were significantly associated with intention to use a modern method. Injunctive norms have been found to positively predict contraceptive intentions among female youth in Niger [37] and sexually-active female adolescents in Addis Ababa [38]; contraceptive use among female youth in Niger [37] and among postpartum women in rural Uganda [39]; and, in a study that applied the IBM, emergency contraceptive use among undergraduate students in the Midwest of the United States [40]. A qualitative study in Nigeria suggested that family, peer and society disapproval of postpartum IUD use negatively affected a woman’s postpartum IUD use intention and behavior, while positive influences from health providers encouraged postpartum IUD use [41].

Out of six studies identified that explored the influence of descriptive norms on contraceptive intentions or use, only two did not find a significant association [36,42]. The remaining studies suggested that descriptive norms were a positive predictor of contraceptive intention or use. For example, Boulay et al. [43] suggested that respondents with favorable descriptive norms were more likely to be using a modern contraceptive method and Wohlwend et al. [40] found that descriptive norms were a positive predictor of intention to use emergency contraception. In India, Rimal et al. [44] found that among women with two or more children, those who perceived more women in the community as using a method of contraception were more likely to use a contraceptive method themselves. In that study, the influence of descriptive norms on contraceptive use was not statistically significant among women with no children and only one child.

Many studies have shown that personal agency is a strong determinant of contraceptive intentions and use; however, findings are sometimes inconsistent. Perceived behavioral control influenced intention to use long-acting reversible contraceptive methods among college women using the contraceptive pill in the United States [45] and contraceptive intentions among rural Ugandan women [39]. In another study, perceived behavioral control was not related to the frequency of condom use [32]. Self-efficacy has also been found to be a strong predictor of contraceptive use. For example, in a survey of women age 18–40 years in Pennsylvania who did not intend to become pregnant for 12 months, prescription contraceptive use was significantly higher among those with high as opposed to low self-efficacy [16]. Studies have also shown that self-efficacy is strongly associated with condom intention and use among adolescents and young people in sub-Saharan Africa [31,46].

The primary objective of this study was to examine the association of perceived norms and personal agency with PPFP intentions among first-time mothers (FTMs) age 15–24 in Kinshasa, the capital city of the DRC. The DRC has one of the highest adolescent birth rates in the world (138 per 1000 live births) and a third of births in the past five years to women age 19 or younger and 28 percent of births to those age 20–24 were wanted later or not at all [47]. A secondary objective was to test the influence of normative expectations, that is, expectations about what other think should be done [48], on PPFP intentions by adding this construct to the IBM. Additional objectives were to determine whether norms exerted the same degree of influence on PPFP intentions across ethnic groups and to better understand the simultaneous influences of injunctive norms and social sanctions for PPFP use by modeling the interactions between them.

The analysis was guided by four hypotheses. Hypothesis one was that injunctive norms and descriptive norms about PPFP use would be positively associated with PPFP intentions. Hypothesis two was that normative expectations about PPFP use would be positively associated with PPFP intentions. Hypothesis three was that personal agency regarding PPFP use would be positively associated with PPFP intentions. As social expectations for women to have many children as soon as possible after marriage are deeply entrenched in some kinship systems [49], it was of interest in our study to examine the extent to which normative expectations were moderated by ethnicity. We were interested in examining the differences between matrilineal groups, where women have traditionally been more dependent on men within the matrilineal kinship system than on men within marriage and sexual relationships, and patrilineal groups [50]. Our fourth hypothesis was that ethnicity would moderate the association between normative expectations and PPFP intentions, with the positive association between these measures being stronger among the Bakongo, a matrilineal ethnic group. We hope that the results of the analysis would help programs move beyond individual-level behavior change and provide evidence to support the design and implementation of PPFP norms-shifting interventions for adolescent girls and young women in Kinshasa.

## Materials and methods

### Data

Data were derived from the 2018 baseline survey for Momentum, a gender-transformative integrated FP, maternal and newborn health and nutrition project conducted among FTMs age 15–24 and their male partners. The sample design was purposive. FTMs were recruited at the health facility and community levels. At the health facility level, the pilot study implementing organization contacted trained prenatal healthcare providers in 11 designated facilities in the health zones of study and asked for their assistance in identifying clients who met the eligibility criteria. Trained prenatal healthcare providers in these designated health facilities introduced the research study to women age 15–24 years who were about six months pregnant with their first child. If the potential subject was interested in study participation, she was (a) given an invitation coupon and instructed to contact the pilot study implementation organization directly or a trained recruiter from that organization who was stationed at the health facility or (b) asked to permit the health care provider to share her interest in study participation with the implementing organization so that a representative could contact her and provide more information about the study. The healthcare provider who introduced the study to the potential subject documented this permission. Only 40 FTMs were recruited at the health facility level. At the community level, trained recruiters employed by a collaborating community-based organization contacted the health zone authorities and community health workers to request their assistance in going house-to-house to identify eligible FTMs.

If the potential research subject was interested in study participation, the recruiter explained the objectives and content of the survey, among other things. The subject was asked if she was willing to be contacted at home for the baseline survey. If the subject agreed, the recruiter assigned her a recruitment number and collected her name, address, phone number, expected delivery/due date, and her preferred dates and times for a pre-visit to ensure that her address could be located and for administering the baseline interview. This information was recorded on a smartphone using an Open Data Kit form. Recruitment stopped once the desired sample size was attained. The sample size was estimated to detect a 10-percentage point difference in timely initiation of postnatal care with 99% confidence and 99% power, assuming an attrition rate of 25% and a design effect of 2.0.

Data collection took place from September to November 2018 in six health zones of Kinshasa: Bumbu, Kingasani, Lemba, Masina I, Matete, and Ndjili. Using a pre-tested questionnaire (see S1 Appendix), data were collected from pregnant women age 15–24. Criteria for inclusion were being 15–24 years and six-months pregnant with the first child; willing and mentally competent to provide informed consent; able to speak French or Lingala; residence in the intervention or comparison health zones (i.e., not living in the study area on a temporary basis, for work, vacation, or other short-term reason). A total of 2,431 FTMs completed the interview. Written informed consent was obtained by trained interviewers from all participants before the interview started. The study received approval from the Tulane University Institutional Review Board and the University of Kinshasa School of Public Health Ethics Committee.

### Variables

#### PPFP intentions

PPFP was defined in the survey as use of a method of contraception in the first six weeks following childbirth, that is, in the immediate postpartum period when unmet need for contraception is greatest [18]. The PPFP intentions scale captured five likely components of the decision-making process: (a) partner discussion of PPFP in the following month; (b) PPFP discussion with a health worker in the following month; (c) going to a health facility, pharmacy or store to get/buy a method of contraception within the first six weeks following childbirth; (d) PPFP use; and (e) PPFP use even if breastfeeding. Responses ranged from “very likely” (coded 1) to “very unlikely” (coded 4) and were reverse coded to create an additive scale of PPFP intentions. The scale had a reliability coefficient of 0.90 (for more descriptive statistics of the index, see S2 Appendix).

#### Attitudes

Instrumental attitudes (beliefs that FP use is associated with certain attributes or outcomes) were measured by the FTM’s rejection of FP myths. Participants were asked to report the level of agreement (strongly agree, agree, disagree, or strongly disagree, coded “1”, “2”, “3”, and “4”, respectively) with eight FP myths and misconceptions (e.g., “Contraceptives are dangerous to women’s health”, etc.). For more detail on the eight myths and misconceptions, see S2 Appendix. The index was constructed as the sum of the responses with higher values representing greater rejection of FP myths (range: 8 to 32; α = 0.80).

#### Injunctive norms

These norms, representing perceptions about what people in a group deemed to be appropriate behavior, were measured by first asking participants to list up to five people who were most important to them, either generally, or when deciding about the use of a method of contraception and to specify these people’s relationship to them. Next, we asked the participant whether each referent would approve or disapprove of her use of a method of contraception within the first six weeks following childbirth. We weighted the responses about each referent’s perceived approval of PPFP with the participant’s motivation to comply with the given referent by multiplying each referent’s perceived approval of PPFP with their motivation to comply. Motivation to comply was based on the following question: “Please tell me whether you strongly agree, agree, disagree or strongly disagree with each of the following statements: When it comes to using contraception within the first six weeks following childbirth, I want to do: What [REFERENT] thinks I should do. The injunctive norm scale ranged from 0 to 20.

#### Perceived community approval of PPFP

In addition, we measured perceptions about the larger community’s approval of PPFP use based on the following question: “If a woman uses a method of contraception within the first six weeks following childbirth, would community members say good things about her, bad things about her, or would they be indifferent?” Our proxy variable consisted of three categories: approve (i.e., good things (reference group)), disapprove (i.e., bad things), and indifferent, and can be interpreted as a measure of social sanction for PPFP use.

#### Descriptive norms

Descriptive norms (normative beliefs about other people’s behavior) were measured by asking participants how many FTMS age 15–24 years in their community they believed used contraceptive methods within the first six weeks following childbirth: all of them, more than half of them, about half of them, less than half of them, or none of them. The response categories “all of them” and “more than half of them” were combined and coded as “1”, with the remaining categories being coded as “0.”

#### Normative expectations

Normative expectations are beliefs about what others think an individual should do. These norms were measured in the survey by asking the FTM whether she strongly agreed, agreed, disagreed, or strongly disagreed with four statements about what most people who were important to her thought she ought to do (for a detailed description of statements, see S2 Appendix). Responses were reverse-coded, and an additive scale created, with higher values representing greater perceived normative expectations about PPFP use. The scale reliability coefficient for the resulting index was 0.86. Unfortunately, the survey did not investigate what would happen if the FTM did not comply with the expectations of most people who were important to her.

#### Perceived control over PPFP use

Perceived control was measured directly by asking participants: “How much control do you believe you have over the use of a method within the first six weeks following childbirth: none at all (reference group), very little control, some control or complete control.”

#### PPFP self-efficacy

Self-efficacy is a subjective assessment of one’s ability to cope with a given situation and is a measure of personal agency. We based our PPFP self-efficacy scale on questions about the level of confidence the FTM had in her ability to perform the seven PPFP-related behaviors within the first six weeks following childbirth (For a detailed description of behaviors, see S2 Appendix). Responses were on a four-point Likert scale: not at all confident (1); not confident (2); confident (3); and extremely confident (4). We constructed a seven-item summative index as an overall measure of PPFP self-efficacy. The index had a Cronbach’s alpha of 0.92 and ranged from 7 to 28. The higher the index, the greater was the level of PPFP self-efficacy.

#### PPFP autonomy

Autonomy may be defined as freedom from external control or influence. Our measure of PPFP autonomy was derived from the following question: “Earlier, you mentioned five people who are most important to you, either generally, or when deciding about use of a method of contraception. If the following people you mentioned did not want you to use a method of contraception within the first six weeks following childbirth, would you still do it?” This question was asked for each of the named referents. Response categories were “yes,” “no,” and “not mentioned.” The resulting index was additive, ranged from 0 to 5, and represented the number of key influencers whose wishes the FTM would not respect if those wishes were against her use of PPFP.

Additional covariates examined were marital status, age, education, exposure to FP messages, household wealth, ethnicity, health zone of residence, whether the pregnancy was unintended, previous use of FP, and exposure to birth spacing and FP counseling in the past six months. Age consisted of two categories: 15–19 (reference group) and 20–24. Education measured the highest level of schooling attended and consisted of three groups: none/primary/secondary incomplete (reference group), secondary complete, and higher. FP message exposure measured the number of information channels from which the participant heard or saw FP messages in the past few months. The FP message exposure index ranged from 0 to 5 or more.

Household wealth was based on an index constructed using principal components analysis from the floor, wall, and roofing materials of the dwelling, availability of electricity, use of improved drinking water sources, type of toilet, and ownership of household items (radio, television, telephone, computer, refrigerator, stove, watch, mobile phone, bicycle, motorcycle, animal-drawn cart, car, and a boat with a motor). The resulting index consisted of 19 items, had a Cronbach’s alpha of 0.64 and was divided into tiers (low (reference group), medium, and high. Ethnicity distinguished Bas Kasai/Kwilu-Kwango (reference group) from Bakongo, Kasai/Katanga, Tanganyika, and others. Health zone of residence consisted of six categories: Bumbu (reference group), Kingasani, Lemba, Masina 1, Matete, and Ndjili.

Unintended pregnancy was a binary variable measuring whether the FTM did not want to get pregnant at the time that she did. Previous use of FP was categorized as never used, traditional methods only, and modern methods. Exposure to birth spacing and/or FP counseling in the past six months was constructed from questions about the content of counseling during (a) home visits by a FP field worker, with the date of the last visit restricted to the past six months; (b) health facility visits for any reason during the past six months; and (c) antenatal care for the current pregnancy. The counseling exposure variable measured whether the FTM was counseled on both topics (i.e., birth spacing and FP), and consisted of three categories: None, one, and both.

### Data analysis

Exploratory factor analysis was performed to assess whether associations existed between components of the PPFP intentions index and how they were grouped. An eigenvalue of 1 was used to determine the number of latent factors for the index and a factor loading of 0.3 was used to retain items in each factor [51,52]. All the items loaded on one factor (eigenvalue = 3.199) and the factor loading of the items ranged from 0.738 and 0.816. The Kaiser-Meyer-Olkin measure of sampling adequacy was 0.845, which provided support for the creation of the composite score of PPFP intentions. As presented in the previous section, all IBM-related composite measures showed acceptable internal consistency (Cronbach’s alpha > 0.70), except for injunctive norms.

Descriptive statistics were presented as means and standard deviations and percentages, as appropriate. For the bivariate tabulations, we examined the association of perceived norms and personal agency with the percentage who responded “very likely” to questions about intentions to discuss, obtain, and use FP methods within the first six weeks following childbirth. Although response categories for these questions were formatted on a four-point scale ranging from “very likely” to “very unlikely”, our measures of PPFP intention were binary and reflected the percentage who were very likely to have the intentions in question. We based our decision to focus on the percentage who gave a “very likely” response on the fact that only 5 percent of women age 15–19 and 11 percent of those age 20–24 in the DRC were currently using a modern method of contraception in 2013–2014 [47]. In the present analysis, the percentage of FTMs who responded, “very likely”, “likely”, “unlikely”, or “very unlikely” to the question about their likelihood of using a method of contraception with in the first six weeks following childbirth was: 12 percent, 56 percent, 24 percent, and 8 percent, respectively. We felt that for the “very likely” response category, PPFP intentions would be more likely to translate into actual PPFP use, at the same time recognizing that translation of behavioral intentions into actual behavior would depend on volitional control and several FP demand and supply factors. It is also worth noting that indices measuring perceived norms and personal agency were divided into terciles (low, medium, and high) for ease of interpretation. Pearson’s chi-square and one-way ANOVA were used to analyze the relationship between the dependent and independent variables of interest. As the sample was purposive, the data could not be weighted.

Two multivariable linear regression models were estimated. The first model included all variables of interest in the regression and the second added interaction terms between a) ethnic group and normative expectations; (b) injunctive norms and perceived community approval of PPFP; and (c) marital status and age group. For each interaction term, tests of joint significance were conducted. Multicollinearity was assessed using variance inflation factors (VIF) greater than 4.0 and found to be insignificant—the mean VIF was 1.6. Of the 2,431 FTMs who completed the interview, 13 (less than one percent) had missing data on one or more variables of interest Therefore, the analysis was based on 2,418 FTMs. Statistical analysis was conducted using STATA software version 15 (StataCorp, College Station, TX) [53].

## Results

### Participants’ characteristics

Table 1 presents the background characteristics of FTMs who were enrolled in the study. At baseline, FTMs were on average 19.8 years old (SD = 4.328) and 75 percent were unemployed (not shown). Slightly more than half (58 percent) were currently married or living with their partner and more than a quarter were never married. A third of FTMs had completed secondary school and eight percent had a level of education that was higher than secondary. Thirty-five percent of FTMs lived in the poorest households. At least half of FTMs participating in the study reported their ethnic affiliation as Bas Kasai/Kwilu-Kwango, or Bakongo. The unintended pregnancy rate was high (82 percent). Almost half of FTMs had never used a method of contraception and 39 percent had ever used a modern method. Half of the sample was not exposed to birth spacing or FP counseling in the past six months; almost a third were counseled on both topics.

**Table 1:** Distribution of first-time mothers age 15–24 by background characteristics, Kinshasa 2018.

Background Characteristics	Total
Current marital status	
Currently married	10.7
Living together	47.6
Engaged/previously married	12.4
Never married	29.3
Level of education	
None/primary/secondary incomplete	59.5
Secondary complete	32.9
Higher	7.6
Household wealth	
Low	35.2
Medium	33.6
High	31.2
Ethnic group	
Bas Kasai & Kwilu-Kwango	37.3
Bakongo	27.5
Kasai, Katanga, Tanganyika	14.9
Other	20.3
Health zone	
Bumbu	12.5
Kingasani	24.7
Lemba	13.9
Masina I	20.5
Matete	11.5
Ndjili	16.9
Unintended pregnancy	
No	18.4
Yes	81.6
Ever use of contraception	
Never used	48.7
Traditional method	12.4
Modern method	38.9
Exposure to birth spacing and/or FP Counseling in the past 6 months	
Neither	53.3
FP or birth spacing	15.1
Both (FP and birth spacing)	31.6
Total	100.0
**Means**	
Number of family planning channels	1.67 (1.51)
N	2,418

() Standard deviation.

Source: Momentum Project Baseline Survey 2018.

We compared the socio-demographic characteristics of our sample with two data sources: (a) the 2013–2014 DRC DHS comprising 100 primiparous women age 15–24 living in Kinshasa; and (b) the 2018 Performance Monitoring for Action (PMA) survey of Kinshasa which covered five of the six health zones included in the Momentum survey and comprised 29 primiparous women age 15–24 (see S3 Appendix). The samples were not directly comparable with Momentum, which had a sample of nulliparous pregnant women age 15–24. However, it was important to show the differences between our sample and those of representative surveys conducted in the DRC, to the extent possible. Note that level of education had to be redefined for the purpose of comparison. Using Pearson chi-square tests, we found significant differences between our sample and the DHS sample. The Momentum sample had significantly more FTMs who were 15–19 years old, living together with their partner, residing in low- and medium-wealth households, and non-users of contraception than the DHS sample. More FTMs in the Momentum sample had secondary-level education and unintended pregnancies than FTMs in the DHS sample. Fisher exact tests indicated that, compared to FTMs in the 2018 PMA survey, more of the Momentum FTMs were 15–19 years old, living together with their partner, residing in the wealthiest households, and had higher rates of unintended pregnancy. As the Momentum sample of women age 15–24 who were about six-months pregnant with their first child was purposive and cannot be weighted, the results of our analysis must be interpreted with caution and cannot be generalized to first-time mothers age 15–24 in Kinshasa.

The Momentum data showed that attitudes towards contraceptive use were somewhat moderate, with the average FTM endorsing four of the eight myths examined (not shown). The mean FP attitude index, which ranged from 8 to 32, was 19.8 (Table 2). The mean injunctive norms index was 10.4 (range: 0–20). Approximately two in five FTMs believed that community members would disapprove of PPFP (39 percent) and only 12 percent of FTMs surveyed believed that more than half of all FTMs age 15–24 years in their community used contraceptive methods within the first six weeks following childbirth. However, normative expectations were supportive of PPFP: the mean index was 11.0 (SD = 2.3) out of a maximum of 16.

**Table 2:** Mean indices of postpartum FP-related attitudes, normative beliefs, personal agency and intentions, and percent distribution of FTMs age 15–24 by perceived control over PPFP decisions, Kinshasa 2018.

Background Characteristics	Range	Mean/Percent
Attitude		
Mean family planning myth rejection index	8–32	19.8 (4.4)
Norms		
Mean injunctive norms index	0–20	10.5 (5.7)
Perceived societal approval of PPFP (%)		
Approval		32.0
Disapproval		38.6
Indifference		29.4
Descriptive norms (%)		11.6
Mean normative expectations index	4–16	11.0 (2.3)
Personal agency		
Perceived PPFP control (%)		
No control		18.4
Very little control		11.1
Some control		24.9
Complete control		45.7
Mean perceived PPFP self-efficacy index	7–28	18.9 (5.2)
Mean PPFP autonomy index	0–5	2.9 (2.3)
Mean PPFP intentions index	5–20	13.5 (3.3)
N		2,418

() Standard deviation.

Source: Momentum Project Baseline Survey 2018.

Perceived control over PPFP decisions was relatively high. Forty-six percent of FTMs believed that they had complete control over the use of a method of contraception within the first six weeks following childbirth. The mean perceived PPFP self-efficacy index was 18.9 (SD = 5.2). Further examination of the components of this index revealed a significant age difference in the FTMs’ level of confidence that they could discuss using a method of contraception within the first six weeks following childbirth with their husband/partner (p = 0.005), with fewer teenage FTMs reporting they were extremely confident (not shown).

The PPFP autonomy index suggested that not all named referents may have an equal influence on the FTM’s behavior and that FTMs may have conditional preferences. For an average of three of the five named referents, FTMs reported that they would still use a method of contraception within the first six weeks following childbirth (the response categories were “no”, “yes”, and “not mentioned”) even if those referents were opposed to the idea.

### Bivariate tabulations

As Table 3 shows, PPFP intentions were low, ranging from 10 to 13 percent. Overall, 13 percent of FTMs stated they were very likely to discuss use of PPFP with their husband/partner next month; 12 percent stated they were very likely to discuss PPFP with a health worker next month; 10 percent stated they were very likely to get/buy a contraceptive method in the postpartum period; 12 percent stated they were very likely to use PPFP; and 12 percent stated they would use PPFP if breastfeeding. The percentage of FTMs who had specific PPFP intentions did not vary significantly by the extent to which FP myths and misconceptions were rejected by the FTM. In contrast, injunctive norms, descriptive norms, normative expectations, perceived control over PPFP and PPFP self-efficacy were significantly associated with the percentage of FTMs who stated that they were very likely to discuss PPFP with their husband/partner or a health worker next month, get/buy a contraceptive method and use it during the first six weeks following childbirth.

**Table 3:** Percentage of first-time mothers age 15–24 who stated they were very likely to have specific postpartum family planning intentions and the mean intention index by age group and selected variables, Kinshasa 2018.

	Percentage who are very likely to:											Mean (SD)	
Variables	Discuss PPFP—Husband/Partner		Discuss PPFP—Health Worker		Get/Buy FP Method in PPP		Use PPFP		Use PPFP if BF		PPFP Intention Index		N
**Attitude (FP myth rejection)**												***	
Low	13.0		11.0		8.9		11.7		11.2		13.0 (3.492)		987
Medium	14.5		14.0		11.4		12.5		12.2		13.5 (3.203)		738
High	12.7		12.3		11.5		13.1		14.3		14.3 (2.947)		693
**Injunctive norms**		***		***		***		***		***		***	
Low	10.1		10.0		6.6		7.6		7.7		12.0 (3.575)		829
Medium	10.7		10.4		9.7		10.8		10.7		14.1 (2.772)		1,360
High	40.6		31.4		28.4		38.4		39.7		15.7 (2.807)		229
**Perceived societal approval of PPFP**		***		***		***		***		***		***	
Approval	19.9		16.8		14.6		17.9		18.2		14.6 (2.884)		773
Disapproval	11.1		9.9		8.8		9.7		10.5		12.7 (3.402)		934
Indifference	9.1		10.5		8.0		9.7		8.6		13.3 (3.259)		711
**Descriptive norms**		***		***		***		***		***		***	
None to half	11.8		10.4		8.9		10.2		10.4		13.4 (2.213)		2,138
More than half	25.0		26.4		22.1		28.2		27.9		14.5 (3.770)		280
**Normative expectations**		***		***		***		***		***		***	
Low	5.4		7.8		4.7		3.9		3.6		11.0 (3.233)		822
Medium	10.6		9.2		7.9		9.0		9.2		14.4 (2.298)		1,271
High	44.3		35.7		34.8		46.5		47.1		16.2 (2.688)		325
**Perceived PPFP control**		***		***		***		***		***		***	
None	5.0		7.4		4.7		4.3		4.1		10.6 (3.122)		444
Little	10.1		9.3		7.8		10.4		9.3		13.0 (3.224)		268
Some	11.3		9.8		8.3		9.8		10.1		13.9 (2.892)		601
Total	18.6		16.3		14.5		17.4		17.7		14.6 (2.848)		1,105
**Perceived PPFP self-efficacy**		***		***		***		***		***		***	
Low	6.0		7.7		5.4		5.3		5.4		11.2 (3.365)		870
Medium	7.5		7.3		6.3		7.5		7.2		14.4 (2.052)		1,020
High	36.7		29.5		26.7		33.3		34.1		15.7 (2.775)		528
**PPFP Autonomy**		***		***		***		***		***		***	
No referent	9.5		7.9		5.7		6.6		6.4		12.2 (3.552)		908
1–4 referents	17.2		16.5		14.9		15.1		14.6		14.0 (2.982)		424
All referents	15.1		14.3		12.6		16.0		16.6		14.5 (2.791)		1,086
Total	13.4		12.3		10.4		12.3		12.4		13.5 (3.300)		2,418

** p < .01

*** p < .001.

BF: Breastfeeding; FP: Family planning; PPFP: Postpartum family planning; PPP: Postpartum period; SD: Standard deviation.

Source: Momentum Project Baseline Survey 2018.

Similarly, perceived societal approval of PPFP use was significantly associated with the percentage of FTMs who intended to discuss PPFP use with their partner and a health worker, get/buy a contraceptive method, or use PPFP. There was very little difference in the prevalence of specific intentions between those who felt that the larger community would disapprove of PPFP use and those who felt that the larger community would be indifferent.

There was generally little difference in the prevalence of each PPFP intention between FTMs in the low category and those in the medium category of each of the following indices: injunctive norms, normative expectations and perceived PPFP self-efficacy. However, as one moved from the medium to the high category, there was a two- to four-fold increase in the percentage of FTMs who stated that they were very likely to discuss use of PPFP with their husband/partner or a health worker next month, to obtain a contraceptive method in the immediate postpartum period, and to use PPFP. For example, the percentage who stated that were very likely to use PPFP increased from four percent to nine percent and 47 percent among those who were in the low, medium and high categories of the normative expectations index, respectively.

Twice as many FTMs stated that they were very likely to discuss use of PPFP next month with their husband/partner and with a health worker, get/buy a contraceptive method in the immediate postpartum period, and use PPFP if they felt that more than half of FTMs age 15–24 in their community used PPFP. For example, the perception that most FTMs age 15–24 in the community used PPFP was associated with 22 percent versus 9 percent prevalence in the intention to get/buy a method of contraception in the six weeks following childbirth. Furthermore, the ratio in the prevalence of specific intentions between those who perceived they had complete control over PPFP use and those who perceived they had none ranged from three to one for intention to get/buy a contraceptive method during the immediate postpartum period to approximately four to one for intention to use PPFP.

### Multivariable analysis

Results of multivariable linear regression models are shown in Table 4 for variables of interest, after adjusting for covariate listed in the footnotes. Model 1 was the base model. Model 2 added interaction terms for (a) ethnicity and normative expectations, (b) injunctive norms and perceptions about the larger community’s approval of PPFP use, and (c) marital status and age group to Model 1. Coefficients for all independent variables are presented in S4 Appendix. The regression results showed that the more FTMs rejected FP myths and misconceptions, the higher was the PPFP intentions index, after controlling for potential confounders (Table 4).

**Table 4:** Results of multivariable linear regression models of postpartum family planning intentions among first-time mothers age 15–24, Kinshasa 2018.

	Mode1 1		Mode1 2	
Variable	Adjusted Coefficient	95% CI	Adjusted Coefficient	95% CI
Attitudes	0.059***	(0.036, 0.082)	0.056***	(0.032, 0.079)
Injunctive norms	0.004	(-0.018, 0.025)	-0.025	(-0.063, 0.013)
Perceived community approval of PPFP				
Approval	-	-	-	-
Disapproval	-0.422***	(-0.668, -0.175)	-1.015***	(-1.578, -0.451)
Indifference	-0.286**	(-0.538, -0.034)	-0.580*	(-1.198, 0.038)
Descriptive norms	0.444***	(0.134, 0.754)	0.461***	(0.151, 0.771)
Normative expectations	0.472***	(0.417, 0.528)	0.383***	(0.305, 0.461)
Perceived control				
No control	-	-	-	-
Very little control	0.439**	(0.057, 0.820)	0.376*	(-0.007, 0.758)
Some control	0.786***	(0.458, 1.114)	0.778***	(0.450, 1.105)
Total control	0.771***	(0.451, 1.091)	0.751***	(0.430, 1.072)
Self-efficacy	0.207***	(0.184, 0.231)	0.205***	(0.182, 0.229)
PPFP autonomy	0.078***	(0.031, 0.124)	0.074***	(0.027, 0.120)
Never married	-0.191*	(-0.412, 0.030)	-0.375**	(-0.662, -0.087)
Age 20–24	0.025	(-0.191, 0.240)	-0.092	(-0.341, 0.157)
Ethnicity				
Bas Kasai/Kwilu-Kwnago	-	-	-	-
Bakongo	0.151	(-0.111, 0.414)	-1.929***	(-3.097, -0.761)
Kasai/Katanga/Tanganyika	0.280*	(-0.016, 0.575)	-0.754	(-2.159, 0.651)
Other	0.021	(-0.247, 0.290)	-1.215*	(-2.494, 0.065)
*Interactions*				
Bankongo * Normative expectations			0.189***	(0.086, 0.292)
Kasai/Katanga, Tanganyika * Normative expectations			0.093	(-0.032, 0.217)
Other * Normative expectations			0.111*	(-0.003, 0.225)
Injunctive norms * Community disapproval			0.053**	(0.009, 0.097)
Injunctive norms * Community indifference			0.023	(-0.027, 0.072)
Never married * Age 20–24			0.431**	(0.008, 0.854)
Constant	2.284***	(1.450, 3.118)	3.874***	(2.733, 5.016)
N	2,418		2,418	
Adjusted R-squared	0.495		0.499	

*** p<0.01

** p<0.05

* p<0.1.

• Reference group.

Regressions control for level of education, FP message exposure, household wealth, unintended pregnancy, previous use of FP, exposure to counseling on birth spacing and/or FP in the past six months, and health zone.

Source: Momentum Project Baseline Survey 2018.

Perceptions of the larger community’s approval of PPFP use were more predictive of PPFP intentions than injunctive norms (that is, perceived approval of PPFP use by people in the FTM’s reference network). The coefficient for injunctive norms was not statistically significant, suggesting that the approval of named referents had no influence on individual FTMs’ PPFP intentions. However, FTMs who perceived that the larger community disapproved of (i.e., “said bad things”; β = -0.422; 95% CI = (-0.668, -0.175)) or was indifferent about PPFP use (β = -0.286; 95% CI = (-0.538, -0.034) had significantly lower PPFP intentions than those who perceived that the larger community approved of (i.e., “said good things” about) PPFP use. Descriptive norms (that is, perceptions of typical PPFP behavior among FTMs age 15–24 in the community) were also a stronger predictor of PPFP intentions than injunctive norms. Women had significantly higher PPFP intentions if they believed that most (that is, more than half or all) as opposed to fewer FTMs in the community used PPFP. Normative expectations about PPFP use, reflecting beliefs about what others think one should do, were associated with a significant increase in PPFP intentions (β = 0.472; 95% CI = (0.417, 0.528), after controlling for all other variables.

Based on conclusions by Ajzen [54], we entered perceived behavioral control and perceived PPFP-related self-efficacy as separate indices in the regression equations. FTMs who perceived they had very little, some or total control over PPFP use had significantly higher PPFP intentions than those who perceived they had no control. A Wald test of the overall significance of the coefficients of perceived control yielded a p-value < .001 (F(3, 2386) = 8.90). The results also showed that the higher was the FTM’s PPFP self-efficacy, the greater was the PPFP intentions index. A unit increase in PPFP self-efficacy was associated with a 0.207 [95% CI = 0.184, 0.231) increase in the PPFP intentions index. Standardized betas (not shown) suggested that PPFP-related self-efficacy and normative expectations accounted for more variance in PPFP intentions (33 percent each, 66 percent overall) than descriptive norms (4 percent), and perceived behavioral control (8 percent).

PPFP autonomy was significantly associated with PPFP intentions, after controlling for covariates. The higher was the PPFP autonomy, the higher was the PPFP index. Standardized betas (not shown) indicated that PPFP autonomy accounted for 5 percent of the variance explained in PPFP intentions. Further analysis indicated that the correlation between PPFP autonomy and injunctive norms was 0.296 and between self-efficacy and perceived behavioral control was 0.480.

Regarding the moderating effects of ethnicity on normative expectations, of injunctive norms on community disapproval, and of age group on marital status, Model 2 shows that the interaction terms were positive for each moderator. Significant interactions were found between normative expectations and Bakongo (β = .189; 95% CI = (0.086, 0.292) and between normative expectations and other ethnic group (β = 0.111; 95% CI = (-0.003, 0.225)). A test of the joint significance of the interaction between ethnicity and normative expectation yielded a p-value of 0.0043 (F(3, 2380) = 4.39), which suggested that normative processes influencing PPFP intention may vary across ethnic groups. The interaction term between perceived community disapproval and injunctive norms was statistically significant at the one-percent level (β = 0.053; 95% CI = (0.009, 0.095). A test of the joint significance of the interaction between perceived community approval of PPFP and injunctive norms yielded a p-value of 0.0497 (F(3, 2380) = 3.00)). The final interaction term indicated that age group modified the association between being never married and PPFP intentions. The negative association between being never married and PPFP intentions (see S4 Appendix) was significantly weaker in the 20–24 than in the 15–19 age group (β = 0.431; 95% CI = (0.008, 0.854)).

As shown in S4 Appendix, education, multi-channel FP message exposure, household wealth, ethnicity, and health zone of residence) were not significantly associated with PPFP intentions. FTMs who had previously used a modern method of contraception had significantly higher PPFP intentions than those who had never used a method. PPFP intentions were also significantly higher among FTMs who had been exposed to either birth spacing or FP counseling than among those who were not counseled on birth spacing or FP in the past six months. The inclusion of previous use of FP and exposure to birth spacing or FP counseling in the regression model did not attenuate the influences of perceived norms and personal agency on PPFP intentions. The proportion of the variance that was accounted for by the model was 50 percent, which was similar with that of many studies conducted in western societies using the TPB [31].

## Discussion

To our knowledge, the current study is one of few to undertake a comprehensive examination of the influence of social norms on PPFP intentions in a low-income setting, using an adaptation of the IBM as a guiding framework. The IBM acknowledges that the degree to which attitude, perceived norms (injunctive and descriptive), and personal agency (perceived control and self-efficacy) influence intentions may vary across behaviors and populations. Our findings showed that among FTMs age 15–24 in Kinshasa, PPFP intentions were primarily determined by instrumental attitudes that were rejecting of FP myths and misconceptions (i.e., of negative outcomes of FP use), descriptive norms, and personal agency (both perceived control and self-efficacy). The associations of attitudes, personal agency and perceived norms with PPFP intentions were stronger than the associations of age, education, household wealth, residence, and other commonly examined social determinants of health.

Our findings pointed to the usefulness of disentangling the different sources of normative influence (see also Mead et al. [55]). We extended the IBM by including normative expectations about PPFP and perceived community approval of PPFP. Among FTMs age 15–24 in Kinshasa, normative expectations and perceived approval of the larger community were more significant determinants of PPFP intentions than the approval of important referent individuals. While it is possible that, in some communities, those who break the norm of non-use of contraception in the immediate PPFP period may face the possibility of social backlash, this is a matter of conjecture and calls for a comprehensive exploration of positive and negative social sanctions for PPFP use by adolescents and young women. The interaction of injunctive norms and perceived societal approval drew from this supposition and was statistically significant. This result suggested that when the surrounding community was perceived to be unsupportive of contraceptive use in the immediate postpartum period, the association between perceived approval of important referents and PPFP intentions may be magnified.

The interaction term between normative expectations and ethnicity was statistically significant, which suggested that normative processes influencing PPFP intention may vary across ethnic groups. This calls for further research on the extent to which PPFP social norms vary across ethnic groups. Such an understanding is important for designing effective cross-cultural interventions to influence PPFP intentions among FTMs age 15–24. The role of ethnicity in shaping intentions and behavior was demonstrated by a study that showed that social norms had varying degrees of influence on the practice of child marriage across communities in Cameroon, making the practice possible, tolerated, appropriate, or obligatory [56]. In large urban settings, inter-ethnic unions tend to be more common than in rural areas, making it critical to have a deeper understanding of the systems of PPFP social norms that exist in Kinshasa.

The study’s findings showed strong support for our hypotheses and the IBM and were congruent with an extensive body of research that has found that social norms are associated with a range of behaviors, although more studies have focused on health-impairing than on health-promoting behaviors, especially among adolescents. For example, Zaleski et al. [57] found that cigarette smoking intentions were significantly correlated with perceived injunctive norms (perceptions of friends’ disapproval of smoking) but not with actual injunctive norms among early adolescents in the United States. In a study of adolescents’ engagement in risky online sexual behavior, both descriptive norms and injunctive norms were significant predictors, but the effect of descriptive norms was stronger and more consistent than the effect of injunctive norms [58]. Peer norms have also been found to be significantly associated with willingness to drink among early adolescents, with the strongest association being found for descriptive norms [59]. In one study, perceived peer norms played a significant role in both health-impairing behaviors such as junk food consumption and sedentary habits and health-promoting behaviors such as fruit and vegetable consumption, particularly when congruent with descriptive parental norms about those behaviors [60]. Our findings partially supported those of a study among newly married churchgoers in Kinshasa, which found that women’s perceptions of injunctive FP norms and descriptive gender equity norms related to childcare responsibilities were significantly associated with intention to use a modern contraceptive method [36] and those of Nsanya et al. [61] that social network support (male partner and/or friends) was a significant determinant of modern contraceptive use among sexually-active women age 15–19 in Tanzania.

A comparison of our findings on personal agency with those of other studies was limited by the fact that self-efficacy had rarely been examined in relation to contraceptive use. Most studies had focused on condom use intentions and condom use, although there is a growing body of research on the measurement of contraceptive self-efficacy in sub-Saharan Africa (see for example, Whiting-Collins et al. [62]). Our findings were consistent with previous studies showing that perceived self-efficacy predicted willingness to use modern contraception in northern Ethiopia [63], contraceptive use intentions in Kenya and Nigeria [64], modern method use in urban Senegal [65], and condom intentions and use in three sub-Saharan sites [31]. There is no agreement in the literature as to whether self-efficacy and perceived behavioral control are conceptually similar or different, and most studies have measured only self-efficacy. Previous studies demonstrated that perceived behavioral control had a weaker contribution to predicted intentions than self-efficacy (see Albarracin et al. [66] for a meta-analysis of 96 studies conducted largely in Europe and the United States). While our conclusions were similar to the latter findings and to the results of a study by Kalolo and Kibusi [33] showing that perceived behavioral control predicted intentions to use condoms among adolescents in rural Tanzania, some studies found that higher perceived control was not associated with condom use intentions [32] or condom use over time [34].

Our study’s unique contributions lie in (a) our focus on adolescent girls and young women regardless of their religious affiliation, nulliparous pregnant women, and PPFP intentions; (b) our estimation of the relative importance of social norms and personal agency; (c) our testing of a comprehensive model (the IBM) that may point to program strategies for increasing FP demand in the immediate postpartum period among adolescent girls and young women; and (d) our extension of the IBM to consider FTMs’ expectations about others’ personal normative beliefs and, implicitly, about the sanctions that may be enforced [67].

## Limitations

As with all research, the current study has some limitations. First, the study was cross-sectional and correlational. As participants were recruited through a non-systematic or non-random sampling scheme, the data cannot be generalized. We did not distinguish between peer norms and family norms. Studies have suggested that among young people, peers may exert a stronger influence on behavioral intentions and actual behavior than family members. Another limitation of the study was that we only measured the expectations of referents that were named but there may have been other groups that could have had expectations that were either consonant or discordant with those of the named referents. We also had no data on negative sanctions to which FTMs could be subject if they failed to fulfill normative expectations about PPFP use or positive sanctions if they conformed to people’s PPFP-related expectations of them.

It is also important to note that the current study investigated perceptions about other FTMs’ PPFP use (descriptive norms) and not their actual use of PPFP. Previous research has shown that social norms can be misperceived. Therefore, it would be important to determine if FTMs overestimated or underestimated the extent of PPFP use by adolescent girls and young women in their communities. Misperceptions could have persisted over time and may have become entrenched. As misperceptions (if they exist) may pressure FTMs with moderate PPFP intentions to refrain from using contraceptive methods in the six weeks following childbirth, it would be important to assess the actual prevalence of PPFP use in communities within Kinshasa. While we focused on the immediate postpartum period, it is worth exploring intentions in the extended postpartum period, which is the one-year period after delivery and is equally critical for the prevention of unintended pregnancy and reduction of the risks of maternal and child mortality.

We did not include knowledge of modern methods of FP in our regression equation because it is assumed to have a direct effect on behavior and not intentions in the IBM, and we found no variance in contraceptive knowledge in our sample. Our regression equations also excluded measures of experiential attitude or affect, defined as an individual’s emotional response to the idea of using a method of contraception in the immediate postpartum period. Rather, we measured instrumental attitude, a cognitive dimension, determined by beliefs about the outcomes of using FP methods. Our proxy measure was non-endorsement of FP myths and misconceptions, which was found to have a significant positive association with PPFP intentions. Finally, although our measures were theoretically informed and based on previous research, they did not represent established metrics.

Despite these limitations, our study highlighted the importance of normative expectations and personal agency for PPFP intentions. It also underscored the importance of distinguishing between different types of norms. Future research should investigate the degree to which perceived norms influence actual PPFP use and the degree to which intentions predict behavior. This may depend on the degree to which PPFP use is under an FTM’s volitional control. Our findings showed that an FTM’s perception of having some or total control over PPFP decisions had a direct positive association with PPFP intentions as did perceived PPFP-related self-efficacy and autonomy, which were encouraging. It would be instructive to replicate the study in representative samples, validate our measures of PPFP-related self-efficacy in the general population of FTMs age 15–24 in Kinshasa, and parse out the associations of perceived peer and family norms. Future research is also needed to better understand what fosters personal agency in PPFP use.

## Program implications

Strong FP intentions are necessary for any intervention that addresses PPFP use. However, addressing PPFP intentions and use in Kinshasa is challenging because, as in South Sudan [49], strong social norms that married women should have as many children as possible before initiating contraceptive use [68] co-exist with negative traditional views of the consequences of closely-spaced pregnancies on children and their mothers [69]. PPFP was not explicitly addressed in the *National Multisectoral Strategic Plan for Family Planning*, *2014–2020* but, recently, the Government drafted guidelines for integrating PPFP into health facility-based services [70]. At the time of writing, these guidelines were under review.

Our study provided evidence for the design of demand creation programs for FP use in the immediate postpartum period by adolescent girls and young women. To translate FP attitudes into specific messages that can be tested and refined before scale-up, it would be useful for programs to determine which FP myths and misconceptions are most associated with PPFP intentions in a given setting, and to target those through the careful design of persuasive communication activities. Tailoring strategies based on marital status and age group may be beneficial, as our results demonstrated that being never married had stronger negative associations with PPFP intentions among FTMs age 15–19 than among those age 20–24.

There is no consensus on social norm change interventions, but an important first step would be to present information on actual PPFP behaviors (e.g., partner discussion of PPFP, proactive discussion of PPFP with a health worker, and PPFP use) in settings where FTMs have underestimated the prevalence of PPFP discussion and use among FTMs in the community. Programs may consider adopting a role model strategy, featuring PPFP ambassadors who share similar cultural experiences and values as FTMs and exemplify positive PPFP behaviors (e.g., discussing use of PPFP with partner, or health worker, and PPFP use), to engage FTMs in communities where PPFP intentions are low or the actual prevalence of PPFP discussion and use is underestimated.

To bring about community-level change in settings where FTMs’ perceptions about community disapproval of PPFP are correct, FP programs should first create safe spaces for community-level reflection and dialogue about PPFP use by FTMs. Engaging communities in critical reflections and encouraging them to root PPFP norms within their own value systems may help shift norms that are against PPFP use by young mothers. Messages on the role of PPFP in fostering healthy timing and spacing of pregnancy and enabling young women’s educational and employment pursuits may help to create positive social norms around FTMs’ PPFP use, especially if presented by a trusted and credible source. Any community efforts to change PPFP norms would require working with a wide range of people and investing in long-term social norm change.

Addressing FTMs’ self-efficacy, perceived control, and autonomy will be critical for increasing PPFP intentions and use. Programs should train and guide pregnant FTMs on how to negotiate FP use in the immediate postpartum period. Strengthening PPFP self-efficacy would also require reducing FTMs’ anxiety about negative outcomes that may result from PPFP discussion and use and using progressive goal-setting approaches during the prenatal period. These interventions may have to be tailored to individual FTMs through clinic- or community-based in-person counseling sessions that address each FTM’s perceptions, beliefs, and concerns. That said, it would be difficult to improve FTMs’ self-efficacy and perceived control over PPFP use without addressing gender and power imbalances in their lives as well as their economic empowerment.

The findings of our study demonstrated that multiple components of the IBM predicted PPFP intentions, making it critical for FP programs to address significant determinants simultaneously. To influence PPFP intentions among pregnant adolescent girls and young women in a sustainable way, individual-and community-level activities must be accompanied by increased allocation of resources by the government to support community-based health workers who deliver PPFP counseling and services and social and behavioral change communication to adolescent girls, young women, and their key influencers.

## Supporting information

S1 Appendix Questions extracted from the Momentum Baseline questionnaire. (DOCX)

S2 Appendix Descriptive statistics of items included in the various indices used in the paper. (DOCX)

S3 Appendix Comparison of Momentum Baseline, 2013–2014 DHS, and 2018 PMA samples of first-time mothers age 15–24. (DOCX)

S4 Appendix Multivariable linear regression results. (DOCX)
