# Supplementary material for: Perceived norms, personal agency, and postpartum family planning intentions among first-time mothers age 15–24 years in Kinshasa: A cross-sectional analysis
Source: PLoS One. 2021 Jul 9;16(7):e0254085. doi: 10.1371/journal.pone.0254085 (PMC8270160; doi:10.1371/journal.pone.0254085)
Supplement: S1 Appendix — (DOCX) [file pone.0254085.s001.docx]

**S1 Appendix. Questions Extracted from the Momentum Baseline Questionnaire**

**Version Date: July 27, 2018**

| **NO.** | **QUESTION AND FILTERS** | | | | | | | | **CODING CATEGORY** | | | | | | | | | | | | | | | | |
| --- | --- | --- | --- | --- | --- | --- | --- | --- | --- | --- | --- | --- | --- | --- | --- | --- | --- | --- | --- | --- | --- | --- | --- | --- | --- |
| 1 | What is the main source of drinking water for members of your household? | | | | | | | | PIPED WATER ........................................................... 01  TUBEWELL OR BOREHOLE ........................................ 02  DUG WELL . .............................................................. 03  WATER FROM SPRING .............................................. 04  RAINWATER .............................................................. 05  TANKER TRUCK ......................................................... 06  CART WITH SMALL TANK .......................................... 07  SURFACE WATER (RIVER/DAM LAKE/POND, STREAM/CANAL/ IRRIGATION CANAL ...................... 08  OTHER ____________________________ 96  (SPECIFY) | | | | | | | | | | | | | | | | |
| 2 | What kind of toilet facility do members of your household usually use? | | | | | | | | FLUSH OR POUR FLUSH TOILET................................. 01  VENTILATED IMPROVED PIT LATRINE........................ 02  PITLATRINE WITH SLAB............................................. 03  PIT LATRINE WITHOUT SLAB/OPEN PIT..................... 04 COMPOSTING TOILET................................................ 05  BUCKE TOILET........................................................... 06  HANGING TOILET/HANGING LATRINE...................... 07  NOFACILITY/BUSH/FIELD......... ........... .................... 08  OTHER ____________________________ 96  (SPECIFY) | | | | | | | | | | | | | | | | |
| 3 | Does your household have:   1. Electricity? 2. A radio? 3. A television? 4. A non-mobile telephone? 5. A computer? 6. A refrigerator? 7. A stove: gas burner or electric stove? | | | | | | | | \|  \| YES \| NO \| \| --- \| --- \| --- \| \| a) ELECTRICITY \| 1 \| 2 \| \| b) RADIO \| 1 \| 2 \| \| c) TELEVISION \| 1 \| 2 \| \| d) NON-MOBILE TELEPHONE \| 1 \| 2 \| \| e) COMPUTER \| 1 \| 2 \| \| f) REFRIGERATOR \| 1 \| 2 \| \| g) STOVE \| 1 \| 2 \| | | | | | | | | | | | | | | | | |
| 4 | Does any member of this household own:   1. A watch? 2. A mobile phone? 3. A bicycle? 4. A motorcycle/scooter? 5. An animal-drawn cart? 6. A car/truck? 7. A boat with a motor? | | | | | | | | \|  \| YES \| NO \| \| --- \| --- \| --- \| \| a) WATCH \| 1 \| 2 \| \| b) MOBILE PHONE \| 1 \| 2 \| \| c) BICYCLE \| 1 \| 2 \| \| d) MOTORCYCLE/SCOOTER \| 1 \| 2 \| \| e) ANIMAL-DRAWN CART \| 1 \| 2 \| \| f) CAR/TRUCK \| 1 \| 2 \| \| g) BOAT WITH MOTOR \| 1 \| 2 \| | | | | | | | | | | | | | | | | |
| 5 | OBSERVE MAIN MATERIAL OF THE FLOOR OF THE DWELLING.  RECORD OBSERVATION. | | | | | | | | **NATURAL FLOOR**  EARTH/SAND ............................................... 11  DUNG .......................................................... 12  **RUDIMENTARY FLOOR**  WOOD PLANKS ....... ............... ..................... 21  PALM/BAMBOO ......... ................................. 22  **FINISHED FLOOR**  PARQUET OR POLISHED WOOD ……….......... 31 VINYL OR ASPHALT STRIPS .......................... 32  CERAMIC TILES ............................................ 33  CEMENT ................ ...................................... 34  CARPET ........... ............................................ 35  OTHER ____________________________ 96  (SPECIFY) | | | | | | | | | | | | | | | | |
| 6 | OBSERVE MAIN MATERIAL OF THE ROOF OF THE DWELLING.  RECORD OBSERVATION. | | | | | | | | **NATURAL ROOFING**  NO ROOF...................................................... 11  THATCH/PALM LEAF .................................... 12  SOD .............................................................. 13  **RUDIMENTARY ROOFING**  RUSTIC MAT ................................................ 21  PALM/BAMBOO ......... ................................ 22  WOOD PLANKS............................................ 23  CARDBOARD ............................................... 24  **FINISHED ROOFING**  METAL ……….............…….......………............. 31  WOOD ............................ ……….................. 32  CALAMINE/CEMENT FIBER ............. ........... 33  CERAMIC TILES ............................................ 34  CEMENT ................ ...................................... 35  ROOFING SHINGLES ..................................... 36  OTHER ____________________________ 96  (SPECIFY) | | | | | | | | | | | | | | | | |
| 7 | OBSERVE MAIN MATERIAL OF THE EXTERIOR WALLS OF THE DWELLING.  RECORD OBSERVATION. | | | | | | | | **NATURAL WALLS**  NO WALLS...................................................... 11  CANE/PALM/TRUNKS.................................... 12  DIRT .............................................................. 13  **RUDIMENTARY WALLS**  BAMBOO WITH MUD .................................. 21  STONE WITH MUD…….................................. 22  PLYWOOD............................................ ....... 24  CARDBOARD ............................................... 25  REUSED WOOD............................................ 26  **FINISHED WALLS**  CEMENT ...................................................... 31  STONE WITH LIME/CEMENT........................ 32  BRICKS ……….............…….......………............. 33  CEMENT BLOCKS ..............……….................. 34  COVERED ADOBE......................................... 35  WOOD PLANKS/SHINGLES ........................... 36  OTHER ____________________________ 96  (SPECIFY) | | | | | | | | | | | | | | | | |
| 8 | In what month and year were you born? | | | | | | | | ┌───┬───┐  MONTH │░░░│░░░│  └───┴───┘  DON’T KNOW MONTH 98  ┌───┬───┬───┬───┐  YEAR │░░░│░░░│░░░│░░░│  └───┴───┴───┴───┘  DON’T KNOW YEAR 9998 | | | | | | | | | | | | | | | | |
| 9 | Have you ever attended school? | | | | | | | | YES 1  NO 2 | | | | | | | | | | | | | | | | |
| 10 | What is the highest level of school you attended: primary, secondary or higher? | | | | | | | | PRIMARY 1  SECONDARY 2  HIGHER 2 | | | | | | | | | | | | | | | | |
| 11 | What is the highest (GRADE/FORM/YEAR) you completed at that level?  IF COMPLETED LESS THAN ONE YEAR AT THAT LEVEL, RECORD '00'. | | | | | | | | ┌───┬───┐  [GRADE/FORM YEAR] │░░░│░░░│  └───┴───┘ | | | | | | | | | | | | | | | | |
| 12 | What is your ethnic group? | | | | | | | | BAKONGO 01  BAS KASAI AND KWILU-KWANGO 02  CUVETTE CENTRAL 03  UBANGIETITIMBIRI-NGIRI 04  UELE; LAKE ALBERT 05  BASELE-KOMO, MANIEMA ET KIVU 06  KASAÏ; KATANGA; TANGANYIKA 07  LUNDA 08  PYGMY 09  OTHER ____________________________ 96  (SPECIFY) | | | | | | | | | | | | | | | | |
| 13 | What is your marital or partnership status now? | | | | | | | | CURRENTLY MARRIED 1  LIVING TOGETHER 2  FORMALLY ENGAGED 3  WIDOWED 4  DIVORCED 5  SEPARATED 6  NEVER MARRIED 7  OTHER ____________________________ 9  (SPECIFY) | | | | | | | | | | | | | | | | |
| 14 | When you got pregnant, did you want to get pregnant at that time? | | | | | | | | YES 1  NO 2 | | | | | | | | | | | | | | | | |
| 15 | Have you ever used anything or tried in any way to delay or avoid getting pregnant? | | | | | | | | YES 1  NO 2 | | | | | | | | | | | | | | | | |
| 16 | Which method have you ever used?  CIRCLE METHOD CODE:  CIRCLE CODE FOR HIGHEST METHOD IN LIST. | | | | | | | | FEMALE STERILIZATION 01  MALE STERILIZATION 02  IUD 03  INJECTABLES 04  IMPLANTS 05  PILL 06  CONDOM 07  FEMALE CONDOM 08  EMERGENCY CONTRACEPTION 09  STANDARD DAYS METHOD 10  LACTATIONAL AMENORRHEA METHOD 11  RHYTHMMETHOD 12  WITHDRAWAL 13  OTHER MODERN METHOD 95  OTHER TRADITIONAL METHOD 96 | | | | | | | | | | | | | | | | |
| 17 | During the last twelve months, has anyone visited you in your house to talk about family planning or to give you family planning methods?  If YES, who came? | | | | | | | | GOVT. FP WORKER A  GOVT. HEALTH WORKER B  MOMENTUM NURSING STUDENT C  NGO FP WORKER D  COMMUNITY-BASED DISTRIBUTOR E  NO ONE F | | | | | | | | | | | | | | | | |
|  |  | | NAME | | | |  | |  |  |  |  |  |  |  |  |  |  |  |  |  |  |  |  |  |
|  | Anyone else? | | | | | | | |  |  |  |  |  |  |  |  |  |  |  |  |  |  |  |  |  |
|  |  | |  | |  |  |  | |  |  |  |  |  |  |  |  |  |  |  |  |  |  |  |  |  |
|  |  | | NAME | | | |  | |  |  |  |  |  |  |  |  |  |  |  |  |  |  |  |  |  |
|  |  | |  | | | |  | |  |  |  |  |  |  |  |  |  |  |  |  |  |  |  |  |  |
|  | WRITE DOWN NAME OF FIELDWORKER | | | | | | | |  |  |  |  |  |  |  |  |  |  |  |  |  |  |  |  |  |
| 18 | When was the last visit? | | | | | | | | ┌───┬───┐  MONTH │░░░│░░░│  └───┴───┘  DON’T KNOW MONTH 98  ┌───┬───┬───┬───┐  YEAR │░░░│░░░│░░░│░░░│  └───┴───┴───┴───┘  DON’T KNOW YEAR 9998 | | | | | | | | | | | | | | | | |
| 19 | Please tell me whether the field worker (CBD) talked about or did the following things:  a) For your and your baby’s health, wait at least 24 months (2 years) after a birth before thinking about becoming pregnant again.  b) Discussing family planning with your husband/partner before delivery.  c) Breastfeeding immediately and exclusively after birth  d) If you do not exclusively breastfeed your baby,  your ability to become pregnant again can return 45 days after you have delivered your baby.  e) Your fertility may return before your next menses.  f) If you choose to use a postpartum family planning method, use one that suits you, your breastfeeding status and your family.  g) Consider the Lactational Amenorrhea Method (LAM) as a family planning choice after the birth of your baby.  h) If you are a Lactational Amenorrhea Method (LAM) user, switch to another modern, family planning method as soon as it ends.  i) Consider postpartum intrauterine contraceptive device (IUD) as family planning choice after the birth of your baby.  j) Discussing family planning with your health worker during your postpartum care visit. | | | | | | | | YES NO  a) WAIT AT LEAST 24 MONTHS 1 2  b) DISCUSS FP WITH HUSBAND/  PARTNER 1 2  c) BREASTFEED IMMEDIATELY AND  EXCLUSIVELY AFTER BIRTH 1 2  d) WHEN MENSES CAN RETURN IF  NOT EXCLUSIVELY BREASTFEEDING 1 2  e) FERTILITY MAY RETURN  BEFORE NEXT MENSES. 1 2  f) USE METHOD THAT SUITS YOU,  BF STATUS, AND FAMILY 1 2  g) CONSIDER USING LAM AFTER  BIRTH OF YOUR BABY 1 2  h) SWITCH TO OTHER MODER FP  METHOD AFTER LAM ENDS 1 2  i) CONSIDER POSTPARTUM IUD 1 2  j) DISCUSS FP WITH HEALTH  WORKER DURING POSTPARTUM  CARE VISIT 1 2 | | | | | | | | | | | | | | | | |
| 20 | In the last 6 months, have you visited a health facility for care for yourself? | | | | | | | | YES 1  NO 2 | | | | | | | | | | | | | | | | |
| 21 | Please tell me whether the staff member at the health facility talked about the following things:  a) For your and your baby’s health, wait at least 24 months (2 years) after a birth before thinking about becoming pregnant again.  b) Discussing family planning with your husband/partner before delivery.  c) Breastfeeding immediately and exclusively after birth  d) If you do not exclusively breastfeed your baby,  your ability to become pregnant again can return 45 days after you have delivered your baby.  e) Your fertility may return before your next menses.  f) If you choose to use a postpartum family planning method, use one that suits you, your breastfeeding status and your family.  g) Consider the Lactational Amenorrhea Method (LAM) as a family planning choice after the birth of your baby.  h) If you are a Lactational Amenorrhea Method (LAM) user, switch to another modern, family planning method as soon as it ends.  i) Consider postpartum intrauterine contraceptive device (IUD) as family planning choice after the birth of your baby.  j) Discussing family planning with your health worker during your postpartum care visit. | | | | | | | | YES NO  a) WAIT AT LEAST 24 MONTHS 1 2  b) DISCUSS FP WITH HUSBAND/  PARTNER 1 2  c) BREASTFEED IMMEDIATELY AND  EXCLUSIVELY AFTER BIRTH 1 2  d) WHEN MENSES CAN RETURN IF  NOT EXCLUSIVELY BREASTFEEDING 1 2  e) FERTILITY MAY RETURN  BEFORE NEXT MENSES. 1 2  f) USE METHOD THAT SUITS YOU,  BF STATUS, AND FAMILY 1 2  g) CONSIDER USING LAM AFTER  BIRTH OF YOUR BABY 1 2  h) SWITCH TO OTHER MODER FP  METHOD AFTER LAM ENDS 1 2  i) CONSIDER POSTPARTUM IUD 1 2  j) DISCUSS FP WITH HEALTH  WORKER DURING POSTPARTUM  CARE VISIT 1 2 | | | | | | | | | | | | | | | | |
| 22 | Please tell me whether you strongly agree, agree, disagree, or strongly disagree with each of the following statements: | | | | | | | | | STRONGLY AGREE | | | AGREE | | DISAGREE | | | | STRONGLY DISAGREE | | | | | | |
|  |  | | | | | | | | |  | | |  | |  | | | |  | | | | | | |
|  | a) | People who use contraceptives end up with health problems. | | | | | | | a) PROBLEMS | 1 | | | 2 | | 3 | | | | 4 | | | | | | |
|  | b) | Contraceptives are dangerous to women's health. | | | | | | | b) DANGEROUS | 1 | | | 2 | | 3 | | | | 4 | | | | | | |
|  | c) | Contraceptives can harm your womb. | | | | | | | c) HARM WOMB | 1 | | | 2 | | 3 | | | | 4 | | | | | | |
|  | d) | Use of a contraceptive injection can make a woman permanently infertile. | | | | | | | d) INFERTILITY | 1 | | | 2 | | 3 | | | | 4 | | | | | | |
|  | e) | Contraceptives reduce women's sexual urge. | | | | | | | e) REDUCED SEX URGE | 1 | | | 2 | | 3 | | | | 4 | | | | | | |
|  | f) | Contraceptives can give you deformed babies. | | | | | | | f) DEFORMED BABIES | 1 | | | 2 | | 3 | | | | 4 | | | | | | |
|  | g) | Women who use family planning may become promiscuous. | | | | | | | g) PROMISCUOUS | 1 | | | 2 | | 3 | | | | 4 | | | | | | |
|  | h) | Contraceptives can cause cancer. | | | | | | | h) CANCER | 1 | | | 2 | | 3 | | | | 4 | | | | | | |
| 23 | Please tell me how likely the following situations are to occur: very unlikely, unlikely, likely, or very unlikely: | | | | | | | | | VERY  LIKELY | | | LIKELY | | UNLIKELY | | | | VERY UNLIKELY | | | | | | |
|  |  |  | | | | | | |  |  | | |  | |  | | | |  | | | | | | |
|  | a) | How likely is it that your husband/partner will **get angry at you** if you said you had to use a method of contraception within the first 6 weeks following childbirth: very unlikely, not likely, likely, or very likely? | | | | | | | a) ANGRY AT YOU | 1 | | | 2 | | 3 | | | | 4 | | | | | | |
|  | b) | How likely is it that your husband/partner will reject you if you said you had to use a method of contraception within the first 6 weeks following childbirth: very unlikely, not likely, likely, or very likely? | | | | | | | b) REJECT YOU | 1 | | | 2 | | 3 | | | | 4 | | | | | | |
|  | c) | How likely is it that your husband/partner] would think you wanted to have sex with someone else if you said you had to use a method of contraception within the first 6 weeks following childbirth: very unlikely, not likely, likely, or very likely? | | | | | | | c) THINK YOU WANT TO HAVE SEX WITH SOMEONE ELSE | 1 | | | 2 | | 3 | | | | 4 | | | | | | |
|  | d) | How likely is it that your husband/partner **stop giving you money** for food and other necessities if you said you had to use a method of contraception within the first 6 weeks following childbirth: very unlikely, not likely, likely, or very likely? | | | | | | | d) STOP GIVING YOU MONEY | 1 | | | 2 | | 3 | | | | 4 | | | | | | |
| 24 | Please tell me up to five people who are most important to you, either generally, or when deciding about use of a method of contraception. What are these people's relationships to you? | | | | | | | | | 1^ST^ | | | 2^ND^ | | 3^RD^ | | 4^TH^ | | | 5^TH^ | | | | | |
|  | a) | Mother? | | | | | | | a) MOTHER | 1 | | | 2 | | 3 | | 4 | | | 5 | | | | | |
|  | b) | Father? | | | | | | | b) FATHER | 1 | | | 2 | | 3 | | 4 | | | 5 | | | | | |
|  | c) | Husband/partner? | | | | | | | c) HUS./PARTNER | 1 | | | 2 | | 3 | | 4 | | | 5 | | | | | |
|  | d) | Sister? | | | | | | | d) SISTER | 1 | | | 2 | | 3 | | 4 | | | 5 | | | | | |
|  | e) | Other family member? | | | | | | | e) OTHER FAMILY | 1 | | | 2 | | 3 | | 4 | | | 5 | | | | | |
|  | f) | Mother-in-law/partner’s mother? | | | | | | | f) MOTHER-IN-LAW | 1 | | | 2 | | 3 | | 4 | | | 5 | | | | | |
|  | g) | Friend? | | | | | | | g) FRIEND | 1 | | | 2 | | 3 | | 4 | | | 5 | | | | | |
|  | h) | Religious authority figure? | | | | | | | h) RELIGIOUS AUTHORITY | 1 | | | 2 | | 3 | | 4 | | | 5 | | | | | |
|  | i) | Health worker? | | | | | | | i) HEALTH WORKER | 1 | | | 2 | | 3 | | 4 | | | 5 | | | | | |
|  | j) | Teacher? | | | | | | | j) TEACHER | 1 | | | 2 | | 3 | | 4 | | | 5 | | | | | |
|  | k) | Co-worker? | | | | | | | k) CO-WORKER | 1 | | | 2 | | 3 | | 4 | | | 5 | | | | | |
|  | l) | Neighbor? | | | | | | | l) NEIGHBOR | 1 | | | 2 | | 3 | | 4 | | | 5 | | | | | |
|  | m) | Other? | | | | | | | m) OTHER | 1 | | | 2 | | 3 | | 4 | | | 5 | | | | | |
|  |  |  | | | | | | |  |  | | |  | |  | |  | | |  | | | | | |
|  |  |  | | (SPECIFY) | | | | |  |  | | |  | |  | |  | | |  | | | | | |
|  | DO NOT READ OUT THE OPTIONS/ RESPONSES. RECORD THE RELATIONSHIP TO FIRST PERSON MENTION IN THE COLUMN "1ST", TO THE SECOND PERSON MENTIONED IN THE COLUMN "2ND", ETC. | | | | | | | | |  |  | |  | |  | |  | |  | | | | |  | |
| 25 | Would the following people you mentioned approve or disapprove of you using a method of contraception within the first six weeks following childbirth? | | | | | | | | | APPROVE | | |  | | DISAPPROVE | |  | | NOT MENTIONED | | | | | | |
|  |  |  | | | | | | |  |  | | |  | |  | |  | |  | | | | |  | |
|  | a) | Mother? | | | | | | | a) MOTHER | 1 | | |  | | 2 | |  | | 3 | | | | |  | |
|  | b) | Father? | | | | | | | b) FATHER | 1 | | |  | | 2 | |  | | 3 | | | | |  | |
|  | c) | Husband/partner? | | | | | | | c) HUSBAND/ PARTNER | 1 | | |  | | 2 | |  | | 3 | | | | |  | |
|  | d) | Sister? | | | | | | | d) SISTER | 1 | | |  | | 2 | |  | | 3 | | | | |  | |
|  | e) | Other family member? | | | | | | | e) OTHER FAMILY | 1 | | |  | | 2 | |  | | 3 | | | | |  | |
|  | f) | Mother-in-law/partner’s mother? | | | | | | | f) MOTHER-IN-LAW | 1 | | |  | | 2 | |  | | 3 | | | | |  | |
|  | g) | Friend? | | | | | | | g) FRIEND | 1 | | |  | | 2 | |  | | 3 | | | | |  | |
|  | h) | Religious authority figure? | | | | | | | h) RELIGIOUS AUTHORITY | 1 | | |  | | 2 | |  | | 3 | | | | |  | |
|  | i) | Health worker? | | | | | | | i) HEALTH WORKER | 1 | | |  | | 2 | |  | | 3 | | | | |  | |
|  | j) | Teacher? | | | | | | | j) TEACHER | 1 | | |  | | 2 | |  | | 3 | | | | |  | |
|  | k) | Co-worker? | | | | | | | k) CO-WORKER | 1 | | |  | | 2 | |  | | 3 | | | | |  | |
|  | l) | Neighbor? | | | | | | | l) NEIGHBOR | 1 | | |  | | 3 | |  | | 3 | | | | |  | |
|  | m) | Other? | | | | | | | m) OTHER | 1 | | |  | | 2 | |  | | 3 | | | | |  | |
|  |  |  | | (SPECIFY) | | | |  |  |  |  | |  | |  | |  | |  | | | | |  | |
|  | CHECK Q5 AND RECORD "3" IF THE PERSON WAS NOT MENTIONED IN Q10. | | | | | | | |  |  |  | |  | |  | |  | |  | | | | |  | |
| 26 | Please tell me whether you strongly agree, agree, disagree, or strongly disagree with each of the following statements:  When it comes to using contraception within the first 6 weeks following childbirth, I want to do: | | | | | | | | | STRONGLY AGREE | | | AGREE | | DISAGREE | | | | STRONGLY DISAGREE | | | | | | |
|  |  |  | | | | | | |  |  | | |  | |  | | | |  | | | | | | |
|  | a) | What my mother thinks I should do. | | | | | | | a) MOTHER | 1 | | | 2 | | 3 | | | | 4 | | | | | | |
|  | b) | What my father thinks I should do. | | | | | | | b) FATHER | 1 | | | 2 | | 3 | | | | 4 | | | | | | |
|  | c) | What my husband/partner thinks I should do. | | | | | | | c) HUSBAND/ PARTNER | 1 | | | 2 | | 3 | | | | 4 | | | | | | |
|  | d) | What my sister thinks I should do. | | | | | | | d) SISTER | 1 | | | 2 | | 3 | | | | 4 | | | | | | |
|  | e) | What other family members think I should do. | | | | | | | e) OTHER FAMILY MEMBERS | 1 | | | 2 | | 3 | | | | 4 | | | | | | |
|  | f) | What my mother-in-law/partner's mother thinks I should do. | | | | | | | f) MOTHER-IN-LAW/PARTNER’S MOTHER | 1 | | | 2 | | 3 | | | | 4 | | | | | | |
|  | g) | What my friends think I should do. | | | | | | | g) FRIENDS | 1 | | | 2 | | 3 | | | | 4 | | | | | | |
|  | h) | What my religion thinks I should do. | | | | | | | h) RELIGION | 1 | | | 2 | | 3 | | | | 4 | | | | | | |
|  | i) | What my health worker thinks I should do. | | | | | | | i) HEALTH WORKER | 1 | | | 2 | | 3 | | | | 4 | | | | | | |
|  | j) | What my teacher thinks I should do. | | | | | | | j) TEACHER | 1 | | | 2 | | 3 | | | | 4 | | | | | | |
|  | k) | What my co-worker thinks I should do. | | | | | | | k) CO-WORKER | 1 | | | 2 | | 3 | | | | 4 | | | | | | |
|  | l) | What my neighbor thinks I should do. | | | | | | | l) NEIGHBOR | 1 | | | 2 | | 3 | | | | 4 | | | | | | |
|  | m) | What people in my community think I should do. | | | | | | | m) COMMUNITY | 1 | | | 2 | | 3 | | | | 4 | | | | | | |
|  | ASK ALL QUESTIONS EVEN IF THE PERSON WAS NOT MENTIONED BY THE RESPONDENT IN Q10. | | | | | | | |  |  |  |  |  |  |  |  | |  |  | |  |  | | |  |
| 27 | Do you approve of women using a method of contraception within the first six weeks following childbirth? | | | | | | | | YES 1  NO 2 | | | | | | | | | | | | | | | | |
| 28 | Do you believe you should start using a method of contraception within the first six weeks following childbirth? | | | | | | | | YES 1  NO 2 | | | | | | | | | | | | | | | | |
| 29 | Do you believe you should start using use a method of contraception within the first six weeks following childbirth, even if you are breastfeeding your baby? | | | | | | | | YES 1  NO 2 | | | | | | | | | | | | | | | | |
| 30 | Do you believe that you should discuss using a method of contraception within the first 6 weeks following childbirth with your husband/partner before the baby is born? | | | | | | | | YES 1  NO 2 | | | | | | | | | | | | | | | | |
| 31 | Do you believe that you can get pregnant if you have sex during the few months following childbirth, even if your menses have not yet returned? | | | | | | | | YES 1  NO 2 | | | | | | | | | | | | | | | | |
| 32 | How many first-time mothers aged 15-24 years in your community do you believe discuss using a method of contraception within the first 6 weeks following childbirth with their husband/partner before the baby is born: all of them, more than half of them, about half of them, less than half of them, or none of them? | | | | | | | | ALL OF THEM 1  MORE THAN HALF OF THEM 2  ABOUT HALF OF THEM 3  LESS THAN HALF OF THEM 4  NONE OF THEM 5 | | | | | | | | | | | | | | | | |
| 33 | How many first-time mothers aged 15-24 years in your community do you believe use contraceptive methods within the first 6 weeks following childbirth: all of them, more than half of them, about half of them, less than half of them, or none of them? | | | | | | | | ALL OF THEM 1  MORE THAN HALF OF THEM 2  ABOUT HALF OF THEM 3  LESS THAN HALF OF THEM 4  NONE OF THEM 5 | | | | | | | | | | | | | | | | |
| 34 | How many first-time mothers aged 15-24 years in your community do you believe use contraceptive methods within the first 6 weeks following childbirth, even if they are breastfeeding their baby: all of them, more than half of them, about half of them, less than half of them, or none of them? | | | | | | | | ALL OF THEM 1  MORE THAN HALF OF THEM 2  ABOUT HALF OF THEM 3  LESS THAN HALF OF THEM 4  NONE OF THEM 5 | | | | | | | | | | | | | | | | |
| 35 | Please tell me whether you strongly agree, agree, disagree, or strongly disagree with each of the following statements: | | | | | | | | | STRONGLY AGREE | | | AGREE | | DISAGREE | | | | STRONGLY DISAGREE | | | | | | |
|  |  |  | | | | | | |  |  | | |  | |  | | | |  | | | | | | |
|  | a) | Most people who are important to me believe that I ought to discuss use of a method of contraception within the first 6 weeks following childbirth with my husband/partner before the baby is born. | | | | | | | a) DISCUSS CONTRACEPTION WITH PARTNER BEFORE CHILDBIRTH | 1 | | | 2 | | 3 | | | | 4 | | | | | | |
|  | b) | Most people who are important to me believe that I ought to start using a method of contraception within the first 6 weeks following childbirth. | | | | | | | b) USE CONTRACEPTION. WITHIN FIRST 6 WEEKS FOLLOWING CHILDBIRTH | 1 | | | 2 | | 3 | | | | 4 | | | | | | |
|  | c) | Most people who are important to me believe that I ought to start using a method of contraception within the first 6 weeks following childbirth, even if I am breastfeeding my baby. | | | | | | | c) USE CONTRACEPTION WITHIN FIRST 6 WEEKS EVEN IF BREASTFEEDING | 1 | | | 2 | | 3 | | | | 4 | | | | | | |
|  | d) | Most people who are important to me believe that women have a right to make family planning decisions. | | | | | | | d) WOMEN HAVE RIGHT TO MAKE FAMILY PLANNING DECISIONS | 1 | | | 2 | | 3 | | | | 4 | | | | | | |
| 36 | How much control do you believe you have over the use of a method of contraception within the first 6 weeks following childbirth: none at all, very little control, some control, or complete control? | | | | | | | | NONE AT ALL 1  VERY LITTLE CONTROL 2  SOME CONTROL 3  COMPLETE CONTROL 4 | | | | | | | | | | | | | | | | |
| 37 | How confident are you that you could discuss using a method of contraception within the first 6 weeks following childbirth with your husband/partner: not at all confident, not confident, confident, or extremely confident? | | | | | | | | NONE AT ALL 1  VERY LITTLE CONTROL 2  SOME CONTROL 3  COMPLETE CONTROL 4 | | | | | | | | | | | | | | | | |
| 38 | How confident are you that you could use a method of contraception within the first 6 weeks following childbirth even if you were afraid that your husband/partner would get angry at you: not at all confident, not confident, confident, or extremely confident? | | | | | | | | NONE AT ALL 1  VERY LITTLE CONTROL 2  SOME CONTROL 3  COMPLETE CONTROL 4 | | | | | | | | | | | | | | | | |
| 39 | How confident are you that you could use a method of contraception within the first 6 weeks following childbirth even if you were afraid that your husband/partner would reject you: not at all confident, not confident, confident, or extremely confident? | | | | | | | | NONE AT ALL 1  VERY LITTLE CONTROL 2  SOME CONTROL 3  COMPLETE CONTROL 4 | | | | | | | | | | | | | | | | |
| 40 | How confident are you that you could use a method of contraception within the first 6 weeks following childbirth even if you were afraid that your husband/partner would think you were having sex with someone else: not at all confident, not confident, confident, or extremely confident? | | | | | | | | NONE AT ALL 1  VERY LITTLE CONTROL 2  SOME CONTROL 3  COMPLETE CONTROL 4 | | | | | | | | | | | | | | | | |
| 41 | How confident are you that you could use a method of contraception within the first 6 weeks following childbirth even if you were afraid that your husband/partner would stop giving you money for food and other necessities: not at all confident, not confident, confident, or extremely confident? | | | | | | | | NONE AT ALL 1  VERY LITTLE CONTROL 2  SOME CONTROL 3  COMPLETE CONTROL 4 | | | | | | | | | | | | | | | | |
| 42 | How confident are you that you can go to a health facility, pharmacy or store to ask for/buy a method of contraception within the first 6 weeks following childbirth, without feeling embarrassed: not at all confident, not confident, confident, or extremely confident? | | | | | | | | NONE AT ALL 1  VERY LITTLE CONTROL 2  SOME CONTROL 3  COMPLETE CONTROL 4 | | | | | | | | | | | | | | | | |
| 43 | If your husband/partner and you were getting "turned on" sexually in the first 6 weeks following childbirth and you could not bring up the subject of protection, how confident are you that you could stop things so that you don't have intercourse: not at all confident, not confident, confident, or extremely confident? | | | | | | | | NONE AT ALL 1  VERY LITTLE CONTROL 2  SOME CONTROL 3  COMPLETE CONTROL 4 | | | | | | | | | | | | | | | | |
| 44 | Please tell me whether it is very unlikely, not likely, likely, or very likely that you will do the following things: | | | | | | | | | VERY  LIKELY | | | LIKELY | | UNLIKELY | | | |  | | | | | | |
|  |  |  | | | | | | |  |  | | |  | |  | | | |  | |  | |  | | |
|  | a) | During the next month, you intend to discuss the use of a method of contraception within first 6 weeks of childbirth with your husband/partner. | | | | | | | a) DISCUSS WITH HUS./PARTNER | 1 | | | 2 | | 3 | | | |  | |  | |  | | |
|  | b) | During the next month, you intend to discuss use of a method of contraception within first 6 weeks of childbirth with a health worker. | | | | | | | b) DISCUSS WITH HEALTH WORKER | 1 | | | 2 | | 3 | | | |  | |  | |  | | |
|  | c) | You intend to go to a health facility, pharmacy, or store to get/buy a method of contraception within the first 6 weeks following childbirth. | | | | | | | c) GET/BUY A METHOD | 1 | | | 2 | | 3 | | | |  | |  | |  | | |
|  | d) | You intend to use a method of contraception within the first 6 weeks following childbirth. | | | | | | | d) USE A METHOD | 1 | | | 2 | | 3 | | | |  | |  | |  | | |
|  | e) | You intend to use a method of contraception within the first 6 weeks following childbirth, even if you are breastfeeding. | | | | | | | e) USE A METHOD EVEN IF BREAST-FEEDING | 1 | | | 2 | | 3 | | | |  | |  | |  | | |
| 45 | Earlier, you mentioned five people who are most important to you, either generally, or when deciding about use of a method of contraception. If the following people you mentioned did not want you to use a method of contraception within the first 6 weeks following childbirth, would you still do it? | | | | | | | | | YES | | | NO | | NOT MEN-TION-ED | | | |  | | | | |  | |
|  | a) | Mother? | | | | | | | a) MOTHER | 1 | | | 2 | | 3 | | | |  | | | | |  | |
|  | b) | Father? | | | | | | | b) FATHER | 1 | | | 2 | | 3 | | | |  | | | | |  | |
|  | c) | Husband/partner? | | | | | | | c) HUSBAND/ PARTNER | 1 | | | 2 | | 3 | | | |  | | | | |  | |
|  | d) | Sister? | | | | | | | d) SISTER | 1 | | | 2 | | 3 | | | |  | | | | |  | |
|  | e) | Other family member? | | | | | | | e) OTHER FAMILY | 1 | | | 2 | | 3 | | | |  | | | | |  | |
|  | f) | Mother-in-law/partner’s mother? | | | | | | | f) MOTHER-IN-LAW | 1 | | | 2 | | 3 | | | |  | | | | |  | |
|  | g) | Friend? | | | | | | | g) FRIEND | 1 | | | 2 | | 3 | | | |  | | | | |  | |
|  | h) | Religious authority figure? | | | | | | | h) RELIGIOUS AUTHORITY | 1 | | | 2 | | 3 | | | |  | | | | |  | |
|  | i) | Health worker? | | | | | | | i) HEALTH WORKER | 1 | | | 2 | | 3 | | | |  | | | | |  | |
|  | j) | Teacher? | | | | | | | j) TEACHER | 1 | | | 2 | | 3 | | | |  | | | | |  | |
|  | k) | Co-worker? | | | | | | | k) CO-WORKER | 1 | | | 2 | | 3 | | | |  | | | | |  | |
|  | l) | Neighbor? | | | | | | | l) NEIGHBOR | 1 | | | 2 | | 3 | | | |  | | | | |  | |
|  | m) | Other? | | | | | | | m) OTHER | 1 | | | 2 | | 3 | | | |  | | | | |  | |
|  |  |  | | (SPECIFY) | | | |  |  |  |  | |  | |  | |  | |  | | | | |  | |
|  | CHECK Q10 AND RECORD "3" IF PERSON WAS NOT MENTIONED. | | | | | | | |  |  |  | |  | |  | |  | |  | | | | |  | |
| 46 | If a woman uses a method of contraception within the first six weeks following childbirth, would community members say good things about her, bad things about her, or would they be indifferent? | | | | | | | | GOOD THINGS 1  BAD THINGS 2  INDIFFERENT 3 | | | | | | | | | | | | | | | | |
| 47 | In the last twelve months, have you seen, heard or read about family planning: | | | | | | | | | YES | | | | | NO | | | |  | | | | | | |
|  |  |  | | | | | | |  |  | | |  | |  | | | |  | | | | | | |
|  | a) | On the radio? | | | | | | | a) RADIO | 1 | | | | | 2 | | | |  | | | | | | |
|  | b) | On the television? | | | | | | | b) TELEVISION | 1 | | | | | 2 | | | |  | | | | | | |
|  | c) | On the internet? | | | | | | | c) INTERNET | 1 | | | | | 2 | | | |  | | | | | | |
|  | d) | From voice or text messages on a mobile phone? | | | | | | | d) MOBILE | 1 | | | | | 2 | | | |  | | | | | | |
|  | e) | In a newspaper or magazine? | | | | | | | e) NEWSPAPER/ MAGAZNE | 1 | | | | | 2 | | | |  | | | | | | |
|  | f) | From a poster/billboard? | | | | | | | f) POSTER | 1 | | | | | 2 | | | |  | | | | | | |
|  | g) | From leaflets and brochures? | | | | | | | g) LEAFLETS | 1 | | | | | 2 | | | |  | | | | | | |
|  | h) | From community events? | | | | | | | h) COMMUNITY EVENTS | 1 | | | | | 2 | | | |  | | | | | | |
|  | i) | From religious leaders speaking in favor of family planning? | | | | | | | i) RELIGIOUS LEADERS | 1 | | | | | 2 | | | |  | | | | | | |
|  | j) | Any other source | | | | | | | j) OTHER | 1 | | | | | 2 | | | |  | | | | | | |
|  |  | SPECIFY | | | | | | |  |  | | |  | |  | | | |  | | | | | | |
| 48 | Are you seeing (did you see) anyone for antenatal care for this pregnancy? | | | | | | | | YES 1  NO 2 | | | | | | | | | | | | | | | | |
|  |  |  |  |  |  |  |  |  |  | | | | | | | | | | | | | | | | |
| 49 | During this pregnancy:   \| a) Were you weighed?  b) Did the health worker feel your abdomen?  c) Was your blood pressure measured?  d) Did you give a urine sample?  e) Did you give a blood sample?   \| f) Were you given or did you buy any iron tablets or syrup?  g) Have you taken SP/Fansidar to prevent you from getting malaria?  h) Did you receive advice on breastfeeding?  i) Did you receive advice on newborn care?  j) Were you advised to sleep under an insecticide-treated net (ITN)?  k) Were you counseled on preparing for birth?  l) Were you counseled on delivering with a skilled birth attendant (SBA)?  m) Were you counseled about birth spacing?  n) Were you counseled on family planning?  o) Were you counseled on prevention of mother-to-child transmission of HIV. \| \| --- \| \| \| \| --- \| --- \| | | | | | | | | \|  \|  \|  \|  \|  \|  \|  \|  \|  \|  \|  \|  \|  \|  \| YES \|  \| NO \| \| --- \| --- \| --- \| --- \| --- \| --- \| --- \| --- \| --- \| --- \| --- \| --- \| --- \| --- \| --- \| --- \| --- \| \|  \|  \|  \|  \|  \|  \|  \|  \|  \|  \|  \|  \|  \|  \|  \|  \|  \| \| a) WEIGHED \| \| \| \| \| \| \| \| \| \| \| \| \| \| 1 \|  \| 2 \| \| b) FELT ABDOMEN \| \| \| \| \| \| \| \| \| \| \| \| \| \| 1 \|  \| 2 \| \| c) BLOOD PRESSURE MEASURED \| \| \| \| \| \| \| \| \| \| \| \| \| \| 1 \|  \| 2 \| \| d) GAVE URINE SAMPLE \| \| \| \| \| \| \| \| \| \| \| \| \| \| 1 \|  \| 2 \| \| e) GAVE BLOOD SAMPLE \| \| \| \| \| \| \| \| \| \| \| \| \| \| 1 \|  \| 2 \| \| f) GIVEN/BOUGHT IRON TABLETS/   SYRUP \| \| \| \| \| \| \| \| \| \| \| \| \| \| 1 \|  \| 2 \| \| g) TAKEN SP/FANSIDAR \| \| \| \| \| \| \| \| \| \| \| \| \| \| 1 \|  \| 2 \| \| h) BREASTFEEDING ADVICE \| \| \| \| \| \| \| \| \| \| \| \| \| \| 1 \|  \| 2 \| \| i) NEWBORN CARE ADVICE. \| \| \| \| \| \| \| \| \| \| \| \| \| \| 1 \|  \| 2 \| \| j) SLEEP UNDER ITN \| \| \| \| \| \| \| \| \| \| \| \| \| \| 1 \|  \| 2 \| \| k) PREPARING FOR BIRTH \| \| \| \| \| \| \| \| \| \| \| \| \| \| 1 \|  \| 2 \| \| l) DELIVERING WITH SBA \| \| \| \| \| \| \| \| \| \| \| \| \| \| 1 \|  \| 2 \| \| m) BIRTH SPACING \| \| \| \| \| \| \| \| \| \| \| \| \| \| 1 \|  \| 2 \| \| n) FAMILY PLANNING \| \| \| \| \| \| \| \| \| \| \| \| \| \| 1 \|  \| 2 \| \| o) PMTCT \| \| \| \| \| \| \| \| \| \| \| \| \| \| 1 \|  \| 2 \| | | | | | | | | | | | | | | | | |
