# Supplementary material for: Perceived norms, personal agency, and postpartum family planning intentions among first-time mothers age 15–24 years in Kinshasa: A cross-sectional analysis
Source: PLoS One. 2021 Jul 9;16(7):e0254085. doi: 10.1371/journal.pone.0254085 (PMC8270160; doi:10.1371/journal.pone.0254085)
Supplement: S2 Appendix — (DOCX) [file pone.0254085.s002.docx]

**S2 Appendix. Descriptive Statistics of Items Included in the Various Indices Used in the Paper**

**S1 Table. Descriptive statistics of items included in the PPFP intention index**

| **PPFP intention Item Index** | **Total** |
| --- | --- |
| **Intention to discuss PPFP use with husband/partner next month** |  |
| Very Unlikely | 9.0 |
| Unlikely | 26.1 |
| Likely | 51.6 |
| Very Likely | 13.4 |
| **Intention to discuss PPFP use with health worker next month** |  |
| Very Unlikely | 6.0 |
| Unlikely | 24.6 |
| Likely | 57.1 |
| Very Likely | 12.3 |
| **Intention to get FP method within 6 weeks of childbirth** |  |
| Very Unlikely | 8.5 |
| Unlikely | 29.4 |
| Likely | 51.7 |
| Very Likely | 10.4 |
| **Intention to use FP within 6 weeks of childbirth** |  |
| Very Unlikely | 7.6 |
| Unlikely | 24.3 |
| Likely | 55.7 |
| Very Likely | 12.3 |
| **Intention to use FP within 6 weeks of childbirth even if breastfeeding** |  |
| Very Unlikely | 8.7 |
| Unlikely | 25.3 |
| Likely | 53.6 |
| Very Likely | 12.4 |
|  |  |
| N | 2,418 |

Source: Momentum Project Baseline Survey 2018

**S2 Table. Descriptive statistics of items included in the instrumental attitude index**

| **Instrumental Attitudes (FP myth rejection) Item Index** | **Total** |
| --- | --- |
| **People who use contraceptives end up with health problems** |  |
| Strongly disagree | 7.7 |
| Disagree | 31.3 |
| Agree | 50.5 |
| Strongly agree | 10.5 |
| **Contraceptives are dangerous to women's health** |  |
| Strongly disagree | 7.9 |
| Disagree | 29.4 |
| Agree | 52.3 |
| Strongly agree | 10.5 |
| **Contraceptives can harm your womb** |  |
| Strongly disagree | 7.7 |
| Disagree | 33.7 |
| Agree | 49.7 |
| Strongly agree | 8.9 |
| **Use of a contraceptive injection can make a woman permanently infertile** |  |
| Strongly disagree | 7.0 |
| Disagree | 29.2 |
| Agree | 51.9 |
| Strongly agree | 11.9 |
| **Contraceptives reduce women's sexual urge** |  |
| Strongly disagree | 13.4 |
| Disagree | 56.4 |
| Agree | 27.0 |
| Strongly agree | 3.2 |
| **Contraceptives can give you deformed babies** |  |
| Strongly disagree | 10.9 |
| Disagree | 52.3 |
| Agree | 31.1 |
| Strongly agree | 5.6 |
| **Women who use family planning may become promiscuous** |  |
| Strongly disagree | 9.1 |
| Disagree | 37.8 |
| Agree | 42.3 |
| Strongly agree | 10.8 |
| **Contraceptives can cause cancer** |  |
| Strongly disagree | 8.5 |
| Disagree | 36.0 |
| Agree | 43.5 |
| Strongly agree | 12.0 |
|  |  |
| N | 2,418 |

Source: Momentum Project Baseline Survey 2018

**S3 Table. Descriptive statistics of items included in the normative expectations index**

| **Normative Expectations Item Index** | **Total** |
| --- | --- |
| **Believes most people think she ought to discuss PPFP with MP before childbirth** |  |
| Strongly disagree | 5.3 |
| Disagree | 26.7 |
| Agree | 57.3 |
| Strongly agree | 10.7 |
| **Believes most people think she ought to start FP use within 6 weeks of childbirth** |  |
| Strongly disagree | 4.8 |
| Disagree | 26.8 |
| Agree | 59.2 |
| Strongly agree | 9.3 |
| **Believes most people think she ought to start FP use within 6 weeks of childbirth** |  |
| Strongly disagree | 5.4 |
| Disagree | 29.5 |
| Agree | 57.2 |
| Strongly agree | 7.9 |
| **Believes most people think women have the right to make FP decisions** |  |
| Strongly disagree | 3.2 |
| Disagree | 18.7 |
| Agree | 65.9 |
| Strongly agree | 12.2 |
|  |  |
| N | 2,418 |

Source: Momentum Project Baseline Survey 2018

**S4 Table. Descriptive statistics of items included in the PPFP self-efficacy index**

| **PPFP Self-efficacy Item Index** | **Total** |
| --- | --- |
| **Discuss PPFP with her husband/partner** |  |
| Not at all confident | 14.1 |
| Not confident | 14.7 |
| Confident | 51.6 |
| Extremely Confident | 19.7 |
| **Use PPFP even if she was afraid that her husband/partner would get angry at her** |  |
| Not at all confident | 14.5 |
| Not confident | 18.1 |
| Confident | 50.5 |
| Extremely Confident | 17.0 |
| **Use PPFP even if she was afraid that her husband/partner would reject her** |  |
| Not at all confident | 13.7 |
| Not confident | 20.1 |
| Confident | 50.2 |
| Extremely Confident | 16.0 |
| **Use PPFP even if she was afraid that her husband/partner would think she was having**  **sex with someone else** |  |
| Not at all confident | 14.8 |
| Not confident | 19.7 |
| Confident | 49.4 |
| Extremely Confident | 16.0 |
| **Use PPFP even if she was afraid that her husband/partner would stop giving her money**  **for food and other necessities** |  |
| Not at all confident | 15.4 |
| Not confident | 18.7 |
| Confident | 50.8 |
| Extremely Confident | 15.1 |
| **Use PPFP even if she was afraid that her husband/partner would go to a health facility,**  **pharmacy or store to ask for/buy a method of contraception without feeling embarrassed** |  |
| Not at all confident | 15.6 |
| Not confident | 17.9 |
| Confident | 50.7 |
| Extremely Confident | 15.9 |
| **Stop things so that she doesn't have intercourse if she and her husband/partner were**  **getting 'turned on' sexually and she could not bring up the subject of protection** |  |
| Not at all confident | 13.1 |
| Not confident | 13.9 |
| Confident | 56.1 |
| Extremely Confident | 16.9 |
|  |  |
| N | 2,418 |

Source: Momentum Project Baseline Survey 2018
