# Supplementary material for: Perceived norms, personal agency, and postpartum family planning intentions among first-time mothers age 15–24 years in Kinshasa: A cross-sectional analysis
Source: PLoS One. 2021 Jul 9;16(7):e0254085. doi: 10.1371/journal.pone.0254085 (PMC8270160; doi:10.1371/journal.pone.0254085)
Supplement: S3 Appendix — (DOCX) [file pone.0254085.s003.docx]

**S3 Appendix. Comparison of Momentum Baseline, 2013-2014 DHS, and 2018 PMA Samples of First-time Mothers Age 15-24**

**S5 Table. Percent distribution of first-time mothers age 15-24 in Kinshasa: 2018 Momentum Baseline Survey, 2013-2014 DHS, and 2018 PMA Survey**

|  | |  |  |  |  |  |  |  |
| --- | --- | --- | --- | --- | --- | --- | --- | --- |
|  | **Momentum vs DHS** | | | |  | **Momentum vs PMA** | | |
|  | **Momentum** | | **DHS** | **p-value ^a^** |  | **Momentum** | **PMA** | **p value ^b^** |
| **Age group** |  | |  | <0.001 |  |  |  | 0.038 |
| 15-19 | 48.1 | | 25.0 |  |  | 48.1 | 27.6 |  |
| 20-24 | 51.9 | | 75.0 |  |  | 51.9 | 72.4 |  |
| **Current marital status** |  | |  | <0.001 |  |  |  | 0.002 |
| Currently married | 10.8 | | 14.0 |  |  | 10.8 | 27.6 |  |
| Living together | 47.6 | | 23.0 |  |  | 47.6 | 31.0 |  |
| Engaged/prev married | 12.4 | | 10.0 |  |  | 12.4 | 0.0 |  |
| Never married | 29.3 | | 53.0 |  |  | 29.3 | 41.4 |  |
| **Level of education** |  | |  | <0.001 |  |  |  | 0.375 |
| None/Primary | 7.2 | | 18.0 |  |  | 7.2 | 13.8 |  |
| Secondary | 85.2 | | 77.0 |  |  | 85.2 | 79.3 |  |
| Higher | 7.6 | | 5.0 |  |  | 7.6 | 6.9 |  |
| **Household wealth** |  | |  | <0.001 |  |  |  | 0.034 |
| Low | 35.2 | | 0.0 |  |  | 35.2 | 48.3 |  |
| Medium | 33.6 | | 0.0 |  |  | 33.6 | 41.4 |  |
| High | 31.2 | | 100.0 |  |  | 31.2 | 10.3 |  |
| **Ethnicity** |  | |  | 0.743 |  |  |  | 0.903 |
| Bas Kasai & Kwilu-Kwango | 37.3 | | 38.0 |  |  | 37.3 | 34.5 |  |
| Bakongo | 27.5 | | 30.0 |  |  | 27.5 | 24.1 |  |
| Kasai, Katanga, Tanganyika | 14.9 | | 11.0 |  |  | 14.9 | 17.2 |  |
| Other | 20.3 | | 21.0 |  |  | 20.3 | 24.1 |  |
| **Ever use of contraception** |  | |  | <0.001 |  |  |  | 0.268 |
| No | 48.7 | | 31.0 |  |  | 48.7 | 37.9 |  |
| Yes | 51.3 | | 69.0 |  |  | 51.3 | 62.1 |  |
| **Unintended pregnancy** |  | |  | <0.001 |  |  |  | <0.001 |
| No | 18.4 | | 72.9 |  |  | 18.4 | 79.3 |  |
| Yes | 81.6 | | 27.1 |  |  | 81.6 | 20.7 |  |
|  |  | |  |  |  |  |  |  |
| Total | 100.0 | | 100.0 | . |  | 100.0 | 100.0 |  |
|  |  | |  |  |  |  |  |  |
| N | 2,418 | | 100 |  |  | 2,418 | 29 |  |
| ^a^ p-values calculated using Pearson’s chi-square test  ^b^ p-values calculated using Fisher's exact test | | | | | | | | |
