# Supplementary material for: Perceived norms, personal agency, and postpartum family planning intentions among first-time mothers age 15–24 years in Kinshasa: A cross-sectional analysis
Source: PLoS One. 2021 Jul 9;16(7):e0254085. doi: 10.1371/journal.pone.0254085 (PMC8270160; doi:10.1371/journal.pone.0254085)
Supplement: S4 Appendix — (DOCX) [file pone.0254085.s004.docx]

**S4 Appendix. Multivariable Linear Regression Results**

**S6 Table. Results of multivariable linear regression models of postpartum family planning intentions among first-time mothers age 15-24, Kinshasa 2018**

|  | **Mode1 1** | |  | **Mode1 2** | |
| --- | --- | --- | --- | --- | --- |
| **Variables** | **Adjusted Coefficient** | **95% CI** |  | **Adjusted Coefficient** | **95% CI** |
| Attitudes | 0.059*** | (0.036, 0.082) |  | 0.056*** | (0.032, 0.079) |
| Injunctive norms | 0.004 | (-0.018, 0.025) |  | -0.025 | (-0.063, 0.013) |
| Perceived community approval of PPFP |  |  |  |  |  |
| Approval | - | - |  | - | - |
| Disapproval | -0.422*** | (-0.668, -0.175) |  | -1.015*** | (-1.578, -0.451) |
| Indifference | -0.286** | (-0.538, -0.034) |  | -0.580* | (-1.198, 0.038) |
| Descriptive norms | 0.444*** | (0.134, 0.754) |  | 0.461*** | (0.151, 0.771) |
| Normative expectations | 0.472*** | (0.417, 0.528) |  | 0.383*** | (0.305, 0.461) |
| Perceived control |  |  |  |  |  |
| No control | - | - |  | - | - |
| Very little control | 0.439** | (0.057, 0.820) |  | 0.376* | (-0.007, 0.758) |
| Some control | 0.786*** | (0.458, 1.114) |  | 0.778*** | (0.450, 1.105) |
| Total control | 0.771*** | (0.451, 1.091) |  | 0.751*** | (0.430, 1.072) |
| Self-efficacy | 0.207*** | (0.184, 0.231) |  | 0.205*** | (0.182, 0.229) |
| PPFP autonomy | 0.078*** | (0.031, 0.124) |  | 0.074*** | (0.027, 0.120) |
| Never married | -0.191* | (-0.412, 0.030) |  | -0.375** | (-0.662, -0.087) |
| Age 20-24 | 0.025 | (-0.191, 0.240) |  | -0.092 | (-0.341, 0.157) |
| Level of education |  |  |  |  |  |
| None/primary/secondary incomplete | - | - |  | - | - |
| Secondary complete | 0.053 | (-0.177, 0.284) |  | 0.048 | (-0.181, 0.278) |
| Higher | -0.093 | (-0.493, 0.308) |  | -0.087 | (-0.486, 0.313) |
| FP message exposure | 0.042 | (-0.025, 0.110) |  | 0.047 | (-0.021, 0.114) |
| Household wealth |  |  |  |  |  |
| Low | - | - |  | - | - |
| Medium | -0.165 | (-0.394, 0.065) |  | -0.192 | (-0.421, 0.038) |
| High | -0.156 | (-0.404, 0.092) |  | -0.164 | (-0.411, 0.083) |
| Ethnicity |  |  |  |  |  |
| Bas Kasai/Kwilu-Kwnago | - | - |  | - | - |
| Bakongo | 0.151 | (-0.111, 0.414) |  | -1.929*** | (-3.097, -0.761) |
| Kasai/Katanga/Tanganyika | 0.280* | (-0.016, 0.575) |  | -0.754 | (-2.159, 0.651) |
| Other | 0.021 | (-0.247, 0.290) |  | -1.215* | (-2.494, 0.065) |
| Unintended pregnancy | -0.163 | (-0.424, 0.098) |  | -0.185 | (-0.447, 0.076) |
| Previous use of family planning |  |  |  |  |  |
| Never used | - | - |  | - | - |
| Traditional method | -0.020 | (-0.327, 0.287) |  | -0.001 | (-0.307, 0.306) |
| Modern method | 0.320*** | (0.106, 0.533) |  | 0.316*** | (0.103, 0.528) |
| Exposure to birth spacing and/or FP counseling | |  |  |  |  |
| None | - | - |  | - | - |
| One | 0.256* | (-0.021, 0.534) |  | 0.249* | (-0.029, 0.526) |
| Both | 0.142 | (-0.077, 0.360) |  | 0.130 | (-0.088, 0.349) |
| Health zone |  |  |  |  |  |
| Bumbu | - | - |  | - | - |
| Kingasani | 0.431** | (0.070, 0.793) |  | 0.407** | (0.046, 0.768) |
| Lemba | 0.192 | (-0.208, 0.591) |  | 0.185 | (-0.214, 0.585) |
| Masina 1 | 0.304 | (-0.068, 0.676) |  | 0.297 | (-0.075, 0.668) |
| Matete | -0.450** | (-0.860, -0.040) |  | -0.489** | (-0.898, -0.079) |
| Ndjili | 0.263 | (-0.105, 0.631) |  | 0.263 | (-0.106, 0.631) |
|  |  |  |  |  |  |
| *Interactions* |  |  |  |  |  |
| Bankongo * Normative expectations |  |  |  | 0.189*** | (0.086, 0.292) |
| Kasai/Katanga, Tanganyika * Normative expectations |  |  |  | 0.093 | (-0.032, 0.217) |
| Other * Normative expectations |  |  |  | 0.111* | (-0.003, 0.225) |
|  |  |  |  |  |  |
| Injunctive norms * Community disapproval |  |  |  | 0.053** | (0.009, 0.097) |
| Injunctive norms * Community indifference |  |  |  | 0.023 | (-0.027, 0.072) |
|  |  |  |  |  |  |
| Never married * Age 20-24 |  |  |  | 0.431** | (0.008, 0.854) |
| Constant | 2.284*** | (1.450, 3.118) |  | 3.874*** | (2.733, 5.016) |
|  |  |  |  |  |  |
| N | 2,418 | |  | 2,418 | |
| Adjusted R-squared | 0.495 | |  | 0.499 | |

*** p<0.01, ** p<0.05, * p<0.1

- Reference group

Source: Momentum Project Baseline Survey 2018
